# Direct Chemical Reprogramming of Human Fibroblasts into Retinal Progenitor-like Cells for Ocular Delivery

**DOI:** 10.3390/jfb17050236

**Published:** 2026-05-08

**Authors:** Yueh-Chang Lee, Pei-Lun Lai, Chien-Ying Lai, Fang-Ling Chang, Shang-Yen Wu, Po-Yu Lin, Chi-Hsuan Chuang, Yu-Xin Chou, Zhao-Feng Chen, Yu-Cheng Wu, Chih-Lun Cheng, Hsuan Lin, Chi-Hou Ng, Shang-Chih Yang, Jean Lu, Rong-Kung Tsai

**Affiliations:** 1 Doctoral Degree Program in Translational Medicine, Tzu Chi University and Academia Sinica, Hualien 970374, Taiwan; josephyclee@mail.harvard.edu (Y.-C.L.);; 2 Department of Ophthalmology, Hualien Tzu Chi Hospital, Buddhist Tzu Chi Medical Foundation, Hualien 970473, Taiwan; 3 Genomics Research Center, Academia Sinica, Taipei 115201, Taiwan; 4 Biomedical Translation Research Center, Academia Sinica, Taipei 115201, Taiwan; 5 Research Center for Applied Sciences, Academia Sinica, Taipei 115201, Taiwan; 6 Institute of Molecular and Cellular Biology, National Taiwan University, Taipei 106319, Taiwan; 7 Institute of Eye Research, Hualien Tzu Chi Hospital, Buddhist Tzu Chi Medical Foundation, Hualien 970473, Taiwan; 8 Institute of Medical Sciences, Tzu Chi University, Hualien 970374, Taiwan

**Keywords:** direct chemical reprogramming, cell engineering, retinal progenitor-like cells, ocular delivery, intravitreal transplantation, subretinal transplantation

## Abstract

Direct chemical reprogramming provides a potentially scalable approach for generating retinal lineage-associated cells without genetic manipulation. In this study, human Tenon’s capsule fibroblasts were converted into retinal progenitor-like cells using a defined small-molecule cocktail. Retinal lineage-associated features were evaluated by immunofluorescence staining, quantitative reverse-transcription PCR, Western blot analysis, and bulk RNA sequencing, showing upregulation of neural and retinal markers, including VSX2, and transcriptomic remodeling consistent with transcriptional features associated with neuronal differentiation programs. Functional responsiveness was assessed by glutamate-evoked intracellular calcium imaging, revealing glutamate-responsive intracellular calcium dynamics in induced cells but not in parental fibroblasts. For in vivo assessment, induced cells were delivered via intravitreal transplantation in Wistar rats and subretinal transplantation in Long–Evans rats. One month after transplantation, structural and functional evaluations using optical coherence tomography, electroretinography, and histological analyses showed localized alterations in retinal structure at the subretinal injection site, while no significant differences were observed in scotopic ERG responses under the present experimental conditions. In contrast, fibroblast transplantation showed more prominent structural alterations under similar conditions. Human nuclei-positive signals were detectable in a subset of eyes, exhibiting focal and heterogeneous distribution within retinal regions at the one-month endpoint. Collectively, these suggest the induction of retinal lineage-associated molecular and functional features, with short-term functional tolerability observed in vivo under the present experimental conditions.

## 1. Introduction

Photoreceptor degeneration represents a common pathological endpoint in a wide spectrum of retinal diseases, including retinitis pigmentosa (RP), diabetic retinopathy (DR), age-related macular degeneration (AMD), and Stargardt’s disease. In advanced stages, these conditions lead to irreversible photoreceptor loss and progressive visual impairment. RP is a major cause of visual deficiency among those younger than middle age, impacting more than 1.5 million individuals worldwide [1]. Out of more than 200 different genotypes in RP, only patients with the *Rpe65* mutation could benefit from gene therapy, which accounts for less than 5% of those with clinically diagnosed RP [2,3]. With the progressive loss of photoreceptor cells, most patients experience visual impairment at night in the early stages of the disease, which may advance to central and color vision decline by midlife [4]. DR is the most common complication of diabetes mellitus (DM), affecting 103 million people worldwide [5]. Clinically, anti-vascular endothelial growth factor therapies, anti-inflammatory agents, and laser interventions may delay disease progression; however, advanced DR accompanied by photoreceptor degeneration remains difficult to manage. AMD is the primary cause of vision impairment among the elderly, affecting nearly 200 million people globally [6]. Once AMD progresses to its late stage with substantial photoreceptor loss, therapeutic options remain limited. Stargardt’s disease, the most common inherited juvenile macular degeneration, affects approximately 1 in 8000–10,000 individuals worldwide [7], most frequently associated with mutations in the *Abca4* gene (also known as *Abcr*) [8]. At present, no clinically established interventions effectively halt this progressive degeneration, and affected individuals often experience significant vision impairment early in life.

Cell-based replacement strategies have been investigated as a potential approach to compensate for photoreceptor loss. Preclinical studies in animal models of RP have demonstrated the feasibility of photoreceptor replacement paradigms and their capacity to support visual function under experimental conditions [9]. While replacement of retinal pigment epithelium (RPE) cells may contribute to slowing disease progression, it does not directly address photoreceptor loss, as RPE cells themselves do not detect light. Clinical investigations using human embryonic stem cells (ESCs) or induced pluripotent stem cells (iPSCs) for RPE replacement have reported structural stabilization and modest functional outcomes in treated patients [10,11,12,13,14,15]. Current strategies targeting photoreceptor restoration can be broadly categorized into three approaches: retinal progenitor cells (RPCs), post-mitotic photoreceptor precursors, and three-dimensional retinal tissue constructs. Among these, RPC-based transplantation has demonstrated encouraging translational potential. To date, 19 preclinical studies have reported the use of human RPCs in models of photoreceptor degeneration (Table S1) [16,17,18,19,20,21,22,23,24,25,26,27,28,29,30,31,32,33,34]. In these studies, RPCs were derived from fetal retina, differentiated from pluripotent stem cells, or generated from multipotent stem cells such as mesenchymal stem cells. Early-phase clinical trials conducted by ReNeuron and jCyte using fetal-derived RPCs in patients with RP (clinicalTrials.gov: NCT02464436; NCT02320812) reported functional stabilization or improvement following transplantation [35,36]. However, practical challenges remain, including surgical complexity associated with high cell delivery volumes [37] and variability in clinical responsiveness depending on baseline retinal status [38].

Despite these advances, scalable and ethically sustainable cell sources remain a critical bottleneck. Fetal-tissue-derived RPCs are limited by availability and ethical considerations, while pluripotent stem cell differentiation protocols are time-intensive, often requiring 30–60 days and complex stage-specific induction procedures [39,40,41]. These constraints highlight the need for alternative cell engineering strategies capable of generating retinal lineage cells in a rapid and reproducible manner.

Direct chemical reprogramming has emerged as a promising approach for lineage conversion without genetic manipulation. In this study, we explored the feasibility of converting adult human Tenon’s fibroblasts (HTFs) into retinal progenitor-like cells using a defined small-molecule cocktail, without exogenous transcription factor delivery. This strategy represents a rapid and conceptually distinct route to retinal lineage induction and provides a potentially translatable platform for generating engineered cellular constructs for ocular delivery applications.

## 2. Materials and Methods

### 2.1. HTFs Isolation and Primary Culture

The samples were obtained anonymously from surgical discard tissues taken during vitrectomy for vitreous hemorrhage, or eye alignment surgery for strabismus from patients. Eligible donors ranged from 7 to 99 years of age. In the present study, tissues from six donors (aged 8, 13, 18, 19, 25, and 28 years) were included (sex: male or female; ethnicity: Taiwanese). The study protocol was approved by the ethical committees of both Hualien Tzu Chi Hospital (IRB107-169-A, IRB109-248-A) and Academia Sinica (AS-IRB-BM-18056, AS-IRB-BM-20056), and adhered to the tenets of the Declaration of Helsinki. Informed consent was obtained from all participants prior to their involvement in the study. We carefully reviewed the donors’ clinical records and excluded those with ocular surface diseases or systemic conditions. During the operations, 0.5 mL of vitreous was collected during vitrectomy, or a 1 × 1 mm^2^ piece of Tenon’s capsule was excised during strabismus surgery. The tissues from vitreous and Tenon’s capsule were cultured separately, and they were promptly transferred to cell culture flasks and maintained in high-glucose DMEM (Life Technologies, Carlsbad, CA, USA, catalog number: 11965092) with 10% fetal bovine serum (FBS) (Peak Serum, Wellington, CO, USA, catalog number: PS-FB2), and 1% penicillin/streptomycin (Life Technologies, Carlsbad, CA, USA, catalog number: 15140122) was added for the first week of culture. IMR-90, BJ-5ta, and CRL-2097 were purchased from ATCC (American Type Culture Collection, Manassas, VA, USA), and FB-3652 (GM03652) was purchased from Coriell (Coriell Institute for Medical Research, Camden, NJ, USA). All fibroblast lines were cultured in DMEM with 10% FBS. Cells were passaged at 80% confluency using TrypLE Express (Gibco, Waltham, MA, USA, catalog number: 12604013) for 5 min at 37 °C. Detached cells were collected, centrifuged at 300× *g* for 5 min, and replated at a 1:3 ratio. HTFs and other fibroblast lines from passages 3 to 10 were used for subsequent experiments.

### 2.2. Flow Cytometry of HTFs

HTFs were collected into 1.5 mL tubes containing Accutase (Thermo Fisher Scientific, Waltham, MA, USA, catalog number: 00-4555-56); then, they were washed three times with cold phosphate-buffered saline (PBS) (Gibco, Waltham, MA, USA, catalog number: 12604013). The cells were permeabilized with ice-cold 90% methanol for 10 min and subsequently stained with an APC-conjugated S100A4 antibody (Bioss Antibodies, Woburn, MA, USA, catalog number: BS-3759R-APC) for 1 h at room temperature. After three additional washes with PBS, the cells were resuspended in FACS buffer (0.1% bovine serum albumin (BSA) (Life Technologies, Carlsbad, CA, USA, catalog number: 15260037) solution in PBS). S100A4^+^ cells were quantified using a BD FACS Canto II flow cytometer (BD Biosciences, Franklin Lakes, NJ, USA).

### 2.3. Preparation of Vsx2::eGFP Promoter Reporter Cells

Reporter cells were generated for HTFs at passage 6 from each donor. To prepare the *Vsx2*::eGFP promoter reporter (Table S2 for the sequences), after PCR amplification, the promoter sequence of *Vsx2* was cloned into pLKO_AS7w.eGFP.puro plasmid (Academia Sinica RNAi core, Taipei, Taiwan), to replace the original *hPGK* promoter fragment. Sanger sequencing was conducted to verify the identity of the pLKO_AS7w.VSX2-eGFP.puro plasmid. The plasmid was then used for lentivirus preparation, following lentivirus production protocol V5 provided by Academia Sinica RNAi core. HTFs were transduced with the prepared lentivirus, following lentivirus infection protocol V4 provided by Academia Sinica RNAi core at a multiplicity of infection (MOI) of 20; 87.0% of the HTFs scored positive for GFP expression with the pLKO_AS7w.eGFP.puro (Figure S1). The reporter cell lines were then used for the experiments described in the following subsections, including the optimization of the reprogramming chemical cocktail and FACS.

### 2.4. Generation of Induced Retinal Lineage-like Cells (iRLCs)

All chemical compounds were diluted in water or DMSO, according to the manufacturer’s datasheet. We generated more than 3 lines of iRLCs for each donor, starting with 1 × 10^6^ HTFs (passage < 10) seeded into a 10 cm dish precoated with Matrigel (1:40 diluted, Corning, Bedford, MA, USA, catalog number: 354234). On day 1, the medium was replaced with HTF medium (10% FBS and DMEM) containing RG108 (20 μM, MedChemExpress, Monmouth Junction, NJ, USA, catalog number: HY-13642). On day 3, the medium was changed to iRLC medium (50% Neural basal (catalog number: 21103049) with 0.075% BSA (catalog number: 15260037), 50% DMEM/F12/Glutamax (catalog number: 10565018), 1X N2 (catalog number: 17502048), 1X B27 without vitamin A (catalog number: 12587010), and 0.1 mM nonessential amino acids (catalog number: 11140050), all from Life Technologies, Carlsbad, CA, USA) with RG108 (20 μM, MedChemExpress, Monmouth Junction, NJ, USA, catalog number: HY-13642) and VPA (3 mM, Tocris Bioscience, Bristol, UK, catalog number: 2815). On day 4, the medium was replaced with iRLC medium (50% Neural basal with 0.075% BSA, 50% DMEM/F12/Glutamax, 1X N2, 1X B27 without vitamin A, and 0.1 mM nonessential amino acids) containing SU9516 (10 μM, R & D Systems, Minneapolis, MN, USA, catalog number: 2907), forskolin (10 μM, Tocris Bioscience, Bristol, UK, catalog number: 1099), Y-27632 (10 μM, LC Laboratories, Woburn, MA, USA, catalog number: Y-5301), and vitamin C (10 μM, Sigma-Aldrich, Saint Louis, MO, USA, catalog number: A7631). On day 5, iRLC medium (50% Neural basal with 0.075% BSA, 50% DMEM/F12/Glutamax, 1X N2, 1X B27 without vitamin A, and 0.1 mM nonessential amino acids) was adjusted to include only forskolin (10 μM), Y-27632 (10 μM), and vitamin C (10 μM). By the end of day 5, the cells had developed a dome shape with bright nuclei and were prone to form clusters, indicating the formation of iRLCs. The cells were observed at the GRC Living Cell System Core Facility with a time-lapse video (images acquired at 30 min intervals), documenting morphological changes during reprogramming. Time-lapse recordings were used for qualitative assessment of cell proliferation and morphological changes during reprogramming. The cells were also observed at BioTrec Automatic Living Cell Assay Core Facility, using the High-Content Screening System, to generate automated quantification of *Vsx2*::eGFP expression. The concentrations and timing of each small molecule used in the chemical reprogramming protocol were determined through a combination of the published literature and the empirical optimization achieved in our experimental system. Karyotyping of passage-10 HTFs, and derived iRLCs was also performed. 1 × 10^6^ HTFs and pairing iRLCs were sent to Ko’s Obstetrics and Gynecology Clinic for clinical-grade chromosome testing. The laboratory uses the international standard G-banding method to perform karyotype analysis on 23 pairs of chromosomes under a microscope to diagnose whether there are numerical or structural abnormalities. This method can detect more than 99% of chromosomal abnormalities. The resolution of G-banding chromosome analysis method is about 3–5 Mb. The presentation of the test results follows the International System for Human Cytogenetic Nomenclature (ISCN) standards. For subculture of iRLCs, the cells were passaged using TrypLE Express (Gibco, Waltham, MA, USA, catalog number: 12604013) for 5 min at 37 °C. Detached cells were collected, centrifuged at 300× *g* for 5 min, and replated at 1:3 ratio. iRLC medium (50% Neural basal with 0.075% BSA, 50% DMEM/F12/Glutamax, 1X N2, 1X B27 without vitamin A, and 0.1 mM nonessential amino acids) with forskolin (10 μM), Y-27632 (10 μM), and vitamin C (10 μM) was replaced every other day.

### 2.5. Immunofluorescence Assay

Cells were treated with 4% formaldehyde (Thermo Fisher Scientific, Waltham, MA, USA, catalog number: J60401.AK) for 15 min at room temperature for fixation and then washed once with 1× PBS. They were permeabilized using 0.3% Triton X-100 (Thermo Fisher Scientific, Waltham, MA, USA, catalog number: A16046.AE) for 5 min, followed by two washes with 1× PBS. Blocking was performed with 2% BSA in PBS for 30 min. The cells were then incubated overnight at 4 °C with primary antibodies: anti-SOX2 (neural stem cell marker; 1:200, GeneTex, Irvine, CA USA, catalog number: GTX101507), anti-OTX2 (anterior neuroepithelium marker; 1:50, R&D, Minneapolis, MN, USA, catalog number: MAB1979), anti-LHX2 (eye field marker; 1:50, Santa Cruz Biotechnology, Dallas, TX, USA, catalog number: SC19344), anti-VSX2 (retinal progenitor cell marker; 1:1000, Novus Biologicals, Centennial, CO, USA, catalog number: NBP184476), and anti-RCVRN (photoreceptor precursor marker; 1:1000, Millipore, Burlington, MA, USA, catalog number: AB5585) in blocking buffer (2% BSA in PBS). After two washes with 1× PBS, the cells were incubated with CF555-conjugated secondary antibodies (1:200, goat anti-mouse (catalog number: A-21422), goat anti-rabbit (catalog number: A-27039), or donkey anti-goat (catalog number: A-21432); all from Life Technologies, Carlsbad, CA, USA) in blocking buffer for 1 h in the dark at room temperature. Nuclei were stained with DAPI dihydrochloride (0.5 μg/mL, Sigma-Aldrich, Saint Louis, MO, USA, catalog number: D9542), and the cells were washed twice with 1× PBS. Fluorescence intensity was quantified using Image-Pro Plus v.4.5 software (Media Cybernetics, Rockville, MD, USA).

### 2.6. Fluorescence-Activated Cell Sorting (FACS) of Induced Retinal Progenitor Cells (iRPCs)

For FACS analysis, iRLCs were dissociated with TrypLE Express (Gibco, Waltham, MA, USA, catalog number: 12604013), filtered through a 40 μm nylon cell strainer (Thermo Fisher Scientific, Waltham, MA, USA, catalog number: 352340), and suspended in PBS with 1% FBS. HTFs were used as the negative control, while iRLCs were analyzed as the sample. Cell sorting was performed using a Becton-Dickinson FACS Aria IIu Flow Cytometer at the GRC core facility. The sorted cells were collected in iRLC medium (50% Neural basal with 0.075% BSA, 50% DMEM/F12/Glutamax, 1X N2, 1X B27 without vitamin A, and 0.1 mM nonessential amino acids) for further applications. The sorted *Vsx2*::eGFP^+^ cells were classified as iRPCs. The purity of the iRPCs population exceeded 90%, with cell viability > 80%, as assessed by immediate post-sort reanalysis.

### 2.7. Real-Time Quantitative Reverse-Transcription PCR (qRT-PCR)

We used TRIzol LS Reagent (Thermo Fisher Scientific, Waltham, MA, USA, catalog number: 10296010) to extract Total RNA, following the manufacturer’s instructions. To eliminate contaminating DNA, the RNA was treated with DNase I (Thermo Fisher Scientific, Waltham, MA, USA, catalog number: EN0521) and then reverse-transcribed with Superscript III (Thermo Fisher Scientific, Waltham, MA, USA, catalog number: 18080093). The resulting cDNAs (100 ng/sample) were used as templates for qRT-PCR with SYBR Green 2X Master Mix (Kapa Biosystems, Wilmington, MA, USA, catalog number: KK4600). The cDNA quantities were measured and quantified using the QuantStudio 5 Real-Time PCR System (Thermo Fisher Scientific, Waltham, MA, USA). The relative levels of target genes were normalized to the RNA levels of *Sdha* (Table S3 for primer sequences).

### 2.8. Western Blot Analysis

Cells were lysed using lysis buffer containing 1% NP-40, 50 mM Tris-HCl (pH 8.0), 150 mM NaCl, 2 mM EDTA, and 1 mM Na_3_VO_4_ supplemented with a protease inhibitor cocktail (Sigma-Aldrich, Saint Louis, MO, USA). Protein concentrations were determined using a Bio-Rad protein assay (Bio-Rad, Hercules, CA, USA). Equal amounts of protein (30 μg per lane) were denatured by boiling at 100 °C for 15 min, separated on 10% SDS–polyacrylamide gels, and transferred onto 0.45 μm nitrocellulose membranes (Amersham Protran). Membranes were blocked with 5% BSA in phosphate-buffered saline with Tween 20 (PBST) for 30 min at room temperature and incubated overnight at 4 °C with primary antibodies against VSX2 (Novus Biologicals, Centennial, CO, USA, catalog number: NBP184476), CRX (Abnova, Taipei, Taiwan, catalog number: H00001406-M06), and β-actin (Sigma-Aldrich, Saint Louis, MO, USA, catalog number: A5441). After washing with PBST, membranes were incubated with appropriate horseradish peroxidase (HRP)-conjugated secondary antibodies for 1 h at room temperature. Protein bands were detected using an enhanced chemiluminescence system and visualized using UVP BioImaging system (Analytik Jena, Thuringia, Germany). Western blot analyses were performed using samples from independent biological replicates derived from different donors. For densitometric analysis, band intensities were quantified using ImageJ software (v1.54r, National Institutes of Health, Bethesda, MD, USA) and normalized to β-actin.

### 2.9. Bulk RNA Sequencing

Total RNA samples from iRPCs at day 6 and parental HTFs were submitted to the Genomics commercial sequencing facility for Bioanalyzer quality control analysis and Illumina Next-Generation Sequencing. All submitted samples had an RNA integrity number (RIN) > 8. Stranded TruSeq cDNA libraries with poly dT enrichment were prepared from total RNA from each sample according to the manufacturer’s protocol. Libraries for the cDNA samples were sequenced using the Illumina HiSeq sequencing platform yielding 24.8–32 million 150 bp paired-end (PE) sequence reads per sample. The sequences of HTFs and FACS-sorted iRPCs were aligned to GRCh37 (hg19). PE FASTQ files received back from Genomics were analyzed using a customized bioinformatics workflow, with mapping rates exceeding 84% (Figure S2). Gene- and transcript-level expressions were quantified using RSEM (RNA-Seq by Expectation–Maximization; http://deweylab.github.io/RSEM/ (accessed on 15 November 2011)), which estimates the expected counts and the normalized expression values while accounting for multi-mapping reads and transcript length. A predefined set of proliferation- and cell cycle-associated genes (e.g., *MKI67*, *MCM* family, *PCNA*, *TOP2A*, *CDK1*, *CCNB1*) was selected based on Gene Ontology annotations and prior literature. Expression patterns of these genes were extracted from RNA-seq data to evaluate changes in proliferative activity during reprogramming. The data have been released in the GEO database (Accession Number: GSE297877).

### 2.10. Calcium Imaging of Glutamate Responses

Intracellular calcium responses to glutamate stimulation were evaluated in parental HTFs, iRLCs, and FACS-sorted iRPCs. Cells were seeded on glass coverslips to enable high-resolution live-cell imaging and loaded with 4 μM fura-2 tetra-acetoxymethyl ester (Thermo Fisher, Waltham, MA, USA, catalog number: F1221) in balanced salt solution (BSS; Alcon, Vernier-Geneva, Switzerland, catalog number: 0065179540) for 40 min at 22 °C. Fluorescence was alternately excited at 340 nm and 380 nm using a filter changer controlled by InCytIM-2 software (v4.50, Intracellular Imaging Corp., Cincinnati, OH, USA) on a phase-contrast microscope (Eclipse T5100; Nikon, Tokyo, Japan). Ratio images (340/380) were captured at 0.35 s intervals. Cells were imaged from 0 to 450 s, and L-glutamate (1 mM) [18] (Sigma-Aldrich, catalog number: 56-86-0) was applied at 100 s. Changes in intracellular calcium dynamics were monitored by assessing alterations in fura-2 fluorescence relative to baseline. Cells were considered responsive based on detectable increases in fluorescence ratio relative to baseline.

### 2.11. Intravitreal Transplantation in Healthy Wistar Rat

Adult Wistar rats were used to evaluate the ocular safety of HTFs and iRLCs following intravitreal delivery. Albino Wistar rats were employed for intravitreal transplantation to facilitate visualization of vitreous cell distribution and retinal interface interactions in the absence of fundus pigmentation. Animals were obtained from the BioLASCO breeding colony (Taipei, Taiwan). All experimental procedures were approved by the Institutional Animal Care and Use Committee of Hualien Tzu Chi Hospital (IACUC109-25).

A single intravitreal injection of 5 μL suspension was administered into the right eye of each animal, containing PBS, HTFs (2 × 10^5^ cells), or iRLCs (2 × 10^5^ cells; reporter-free and without exogenous genetic modification). Each treatment group consisted of six rats (*n* = 6), and each animal was considered an independent experimental unit. Transplanted cells were generated from independent reprogramming batches and were randomly allocated to animals without pooling prior to injection (*n* = 6 biological replicates).

Ocular structural integrity and safety were evaluated one month after injection using fundus photography, optical coherence tomography (OCT), electroretinogram (ERG), and histological analysis to assess retinal morphology, potential structural alterations, functional tolerability at the one-month time point, and short-term graft persistence.

### 2.12. Subretinal Transplantation in Healthy Long–Evans Rat

Subretinal transplantation was performed in healthy adult Long–Evans rats to assess the safety and retinal tolerability of HTFs and iRLCs following subretinal delivery. Pigmented Long–Evans rats were selected to enable stable structural assessment of graft localization within the retinal layers, as fundus pigmentation improves OCT layer discrimination and histological interpretation. Animals were obtained from the BioLASCO breeding colony (Taipei, Taiwan). All procedures adhered to the ARVO Statement for the Use of Animals in Ophthalmic and Vision Research and were approved by the Institutional Animal Care and Use Committee of Hualien Tzu Chi Hospital (IACUC109-44).

Rats were anesthetized with intramuscular ketamine (40 mg/kg) and xylazine (4 mg/kg), with additional topical anesthesia applied. Following pupillary dilation, a subretinal injection was performed by creating a scleral entry site using a 31-gauge needle. A 5 μL suspension was delivered into the subretinal space of the right eye of each animal, containing PBS, HTFs (2 × 10^5^ cells), or iRLCs (2 × 10^5^ cells; reporter-free and without exogenous genetic modification). Each treatment group included six rats (*n* = 6), and each animal was considered an independent experimental unit. Transplanted cells were generated from independent reprogramming batches (*n* = 6 biological replicates). Cells from distinct reprogramming batches were randomly allocated to individual animals rather than pooled prior to transplantation.

Successful transplantation was confirmed by formation of a subretinal bleb without evident reflux, verified by fundus photography and OCT. Ocular structural integrity and safety were evaluated one month after transplantation using fundus imaging, OCT, ERG, and histological analysis to assess retinal morphology, potential structural alterations, functional tolerability at the one-month time point, and short-term graft persistence following subretinal cell delivery.

### 2.13. Optical Coherence Tomography

OCT imaging was performed using contact lenses mounted on a Micron IV retinal imaging system (Phoenix Research Labs, Pleasanton, CA, USA). After anesthesia as described above, pupils were dilated and corneal surfaces were protected with 2% Methocel (OmniVision, Santa Clara, CA, USA; catalog number: 04682367). Animals were positioned on a horizontal platform to ensure perpendicular optical alignment with the cornea. The fundus was visualized using the Micron IV camera, and horizontal OCT scans were acquired through the transplantation site to evaluate retinal layer integrity and structural morphology. At least two scans were obtained per eye. Image acquisition and analysis were performed by investigators masked to treatment allocation.

### 2.14. Electroretinogram

Scotopic and photopic full-field ERG recordings were performed to evaluate retinal physiological integrity following ocular cell delivery. Rats were dark-adapted for at least 24 h prior to anesthesia. After pupil dilation, animals were positioned in a ColorDome Ganzfeld stimulator connected to the Espion Visual Electrophysiology System (Diagnosys LLC, Lowell, MA, USA). ERGs were recorded using contact lens electrodes placed on the corneal surface with 2% Methocel (OmniVision, Santa Clara, CA, USA; catalog number: 04682367) serving as the conductive interface. A reference electrode was inserted subcutaneously at the midline scalp, and a ground electrode was positioned at the proximal tail. Animals were maintained on a heating pad throughout the procedure, and electrode impedance was monitored to ensure signal stability within acceptable limits. For scotopic (dark-adapted) recordings, stimuli were set at 0.01 cd·s/m^2^, and responses from five consecutive flashes were averaged to generate representative waveforms. For photopic (light-adapted) recordings, rod responses were saturated using background illumination of 20 cd·s/m^2^ for at least 10 min prior to light-adapted stimulation at 3.0 cd·s/m^2^. Ten responses were averaged to obtain the final photopic waveform.

ERG recordings were conducted in animals receiving intravitreal or subretinal transplantation as part of the ocular safety and functional tolerability assessment. The mean scotopic b-wave amplitude was used as the primary functional parameter for comparison across treatment groups, consistent with previous studies on RPC transplantation in rats (Table S4) [24,25,26,30,42]. Waveform acquisition and analysis were performed by investigators masked to treatment allocation.

### 2.15. Histological Examination

At the designated endpoint, rats were euthanized by carbon dioxide inhalation, and eyes were enucleated and fixed in 4% paraformaldehyde for 48 h at 4 °C. Following fixation, tissues were cryoprotected in graded sucrose solutions (10%, 20%, and 30%; Thermo Fisher, Waltham, MA, USA; catalog number: A15583.36) overnight at 4 °C. Eyes were embedded in Tissue-Tek compound (Sakura Finetek, Torrance, CA, USA; catalog number: 4583), and 20 μm cryosections were prepared using a Leica CM-3050-S cryostat (Leica, Oberkochen, Germany) and mounted on glass slides.

For general retinal morphology assessment, sections were stained with hematoxylin–eosin (Sakura Finetek, Torrance, CA, USA; catalog number: 6190). For immunofluorescence analysis, sections were permeabilized with 0.3% Triton X-100 for 5 min, washed in PBS, and blocked with 2% BSA in PBS for 30 min. Slides were incubated overnight at 4 °C with primary antibodies against human nuclei (anti-human nuclei, 1:20, Sigma-Aldrich, Saint Louis, MO, USA; catalog number: MAB1281) to identify transplanted human cells. After washing, sections were incubated for 1 h at room temperature in the dark with secondary antibodies (CF555 goat anti-mouse, 1:200, Life Technologies, Carlsbad, CA, USA; catalog number: A-21422) along with DAPI (0.5 μg/mL; Sigma-Aldrich, Saint Louis, MO, USA; catalog number: D9542) for nuclear counterstaining. Slides were mounted using ProLong Gold antifade reagent (Thermo Fisher, Waltham, MA, USA; catalog number: P10144). Fluorescence images were initially examined using an Axiovert 200 M fluorescence microscope (Zeiss, Oberkochen, Germany) for general screening and identification of regions of interest. High-resolution images presented in the figures were acquired using a Leica TCS SP8 confocal laser scanning microscope (Leica, Oberkochen, Germany). Z-stack imaging and optical sectioning were performed to minimize out-of-focus fluorescence and improve nuclear localization accuracy.

Histological evaluation was performed to assess retinal layer integrity, cellular localization, and short-term graft persistence following ocular delivery with particular attention to localized changes in retinal layer organization at or near the injection site. To assess the presence of transplanted human cells, a semi-quantitative approach was adopted. All available sections were initially screened under light microscopy to assess retinal structure. For subretinal transplantation experiments, regions corresponding to the injection site were identified based on anatomical landmarks guided by OCT. Sections encompassing these regions were selected for analysis. For intravitreal transplantation experiments, where a discrete injection site could not be reliably identified, sections were selected at regular intervals across the globe to represent overall retinal regions, rather than based on HuNu signal intensity. From each eye, two sections were selected for subsequent HuNu immunofluorescence analysis, based on anatomical relevance to the injection region rather than signal intensity. An eye was considered “HuNu-positive” if at least one HuNu-positive cell was detected in any examined section. To minimize false-positive interpretation, HuNu-positive signals were required to exhibit clear nuclear morphology and localization distinct from background autofluorescence. Due to the sparse and heterogeneous distribution of transplanted cells, absolute cell counts were not performed. Instead, results are reported as the proportion of eyes with detectable HuNu-positive signals. Image acquisition and analysis were conducted by investigators masked to treatment allocation.

### 2.16. Statistical Analysis

For descriptive statistics, values are expressed as mean ± SD as indicated in the figure legends. All statistical analyses were performed using GraphPad Prism (v10.1.2, GraphPad Software, Boston, MA, USA).

For in vitro experiments, reporter assays involving multiple treatment conditions were analyzed using one-way analysis of variance (ANOVA) followed by Dunnett’s multiple comparisons test versus the 6C control. For qRT-PCR analyses, relative expression was calculated using the 2^−ΔΔCt^ method (shown as fold change for interpretability), whereas statistical testing was performed on ΔCt values. Differences between two groups were evaluated using the Mann–Whitney U test, and *p*-values were adjusted using the Benjamini–Hochberg false discovery rate (FDR) method within each predefined gene panel. For Western blot densitometry, band intensities were normalized to β-actin, and differences between groups were evaluated using an unpaired two-tailed Student’s *t*-test. For bulk RNA sequencing, transcriptomic analyses were exploratory in nature. Genes showing differential expression patterns were further subjected to pathway enrichment and Gene Ontology analyses using curated databases, with enrichment significance determined using FDR-adjusted *p*-values.

For animal studies (Wistar and Long–Evans rats), transplantation experiments were conducted in single independent cohorts (*n* = 6 per group). Between-group comparisons were performed using the Kruskal–Wallis test followed by Dunn’s multiple comparisons test. The individual animal was considered the unit of analysis.

## 3. Results

### 3.1. Primary Culture of Tenon’s Capsule–Derived Fibroblasts

**Figure 1:**
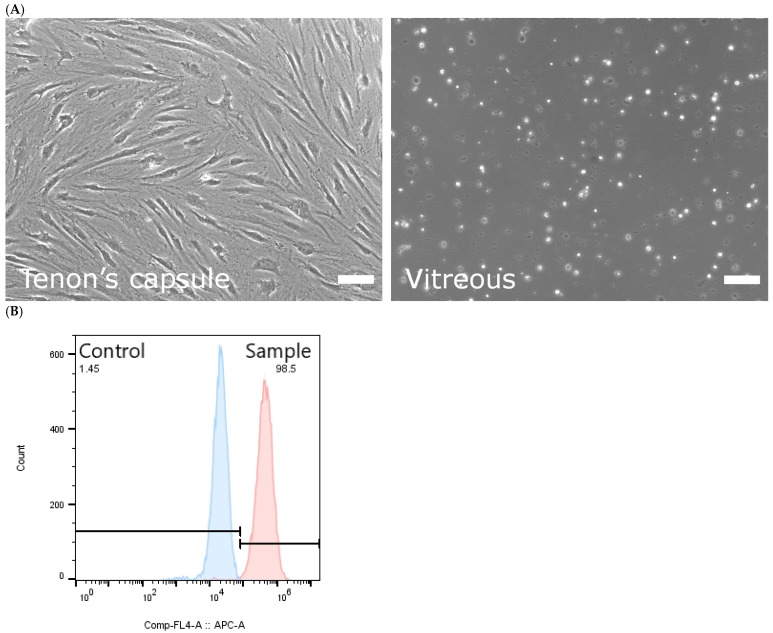
Primary culture and characterization of HTFs. (**A**) Phase-contrast images of primary cultures derived from vitreous samples and Tenon’s capsule tissue. Vitreous cultures showed only cellular debris, whereas Tenon’s capsule consistently generated adherent spindle-shaped fibroblast-like cells. (**B**) Flow cytometric analysis of S100A4 expression in Tenon’s capsule–derived cells. Gating was established based on isotype control staining. The majority of cultured cells (98.5%) were positive for S100A4. Scale bar: 100 μm. Images are representative of cultures derived from six independent donors (*n* = 6).

Donated ocular tissues were obtained during routine eye surgeries. Primary cultures were initiated from both vitreous samples and Tenon’s capsule tissue (tissues from six donors aged 8, 13, 18, 19, 25, and 28 years were included for analysis). Vitreous-derived cultures yielded only cellular debris and did not generate adherent proliferative cells under the culture conditions used. In contrast, Tenon’s capsule samples consistently produced adherent, spindle-shaped or polygonal fibroblast-like cells within one week of culture (Figure 1A). These cells exhibited the characteristic flattened morphology typical of fibroblasts.

To further characterize the cultured cells, flow cytometry was performed for S100 calcium-binding protein A4 (S100A4), a commonly used fibroblast-associated marker. The majority of cultured Tenon’s capsule–derived cells were positive for S100A4 expression (98.5%), supporting their fibroblast identity (Figure 1B). These cells were designated as HTFs and were used for subsequent reprogramming and transplantation experiments.

### 3.2. Generation of Vsx2::eGFP Reporter System to Optimize Combination of Chemical Cocktail

**Figure 2:**
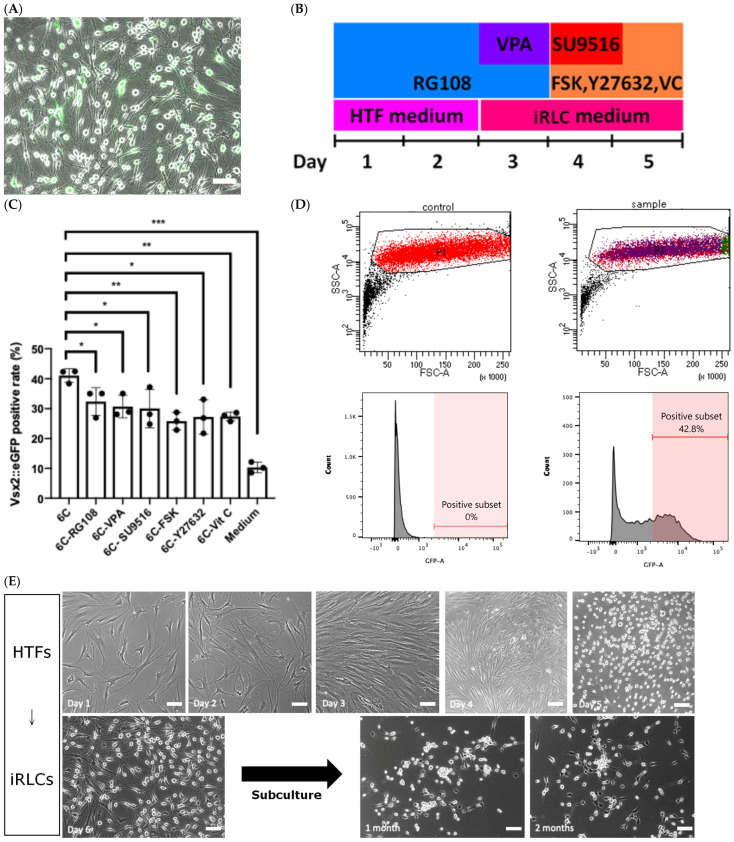
Direct reprogramming of HTFs into iRLCs. (**A**) Representative phase-contrast and fluorescence images showing morphological transition and induction of Vsx2::eGFP expression during the reprogramming protocol. (**B**) Schematic overview of the 5-day 6C reprogramming protocol (RG108, VPA, SU9516, FSK, Y-27632, VC). (**C**) Quantification of *Vsx2*::eGFP-positive cells following systematic omission of individual compounds from the 6C protocol. Data are presented as mean ± SD. Statistical comparisons were performed on independent biological replicates derived from different donors (*n* = 3), using one-way ANOVA followed by Dunnett’s multiple comparisons test versus the 6C condition. ns: not significant; * *p* < 0.05; ** *p* < 0.01; *** *p* < 0.001. (**D**) Representative flow cytometry gating strategy for identification of *Vsx2*::eGFP-positive cells. (**E**) Phase-contrast images illustrating morphological progression from HTFs to iRLCs during and after reprogramming. Cells maintained stable morphology after day 6 and remained viable following subculture for up to two months. Scale bar: 100 μm. Images are representative of cultures derived from six independent donors (*n* = 6).

To optimize the chemical reprogramming strategy, HTFs were transduced with a *Vsx2*::eGFP reporter construct to enable quantitative monitoring of retinal progenitor-associated gene activation. Changes in cell morphology together with induction of reporter expression were used as readouts to evaluate different combinations of small molecules and culture media (Figure 2A).

Since 2014, several groups have reported the chemical induction of neural-lineage cells, including chemical-induced neural progenitor cells described by Cheng et al. [43], neural stem cell–like cells described by Zhang et al. [44], and chemical-induced neurons described by Li et al. [45], Hu et al. [46], and Zhang et al. [47]. In our previous work, we performed high-throughput screening of over 1500 small molecules from Selleckchem and successfully reprogrammed fibroblasts into oligodendrocyte-like cells [48]. Building upon these studies, we tested combinations of compounds reported in neural reprogramming protocols using automated high-content imaging analysis. Through iterative optimization, we identified a six-compound (6C) combination consisting of RG108, valproic acid (VPA), SU9516, forskolin (FSK), Y-27632, and vitamin C (VC), administered over a 5-day protocol (Figure 2B). RG108, a non-nucleoside DNA methyltransferase (DNMT) inhibitor, and VPA, a histone deacetylase inhibitor, were applied during the initial phase to facilitate epigenetic remodeling [49]. SU9516, a cyclin-dependent kinase 2 inhibitor, was subsequently introduced to modulate cell cycle dynamics during lineage conversion [50]. Y-27632 (a ROCK inhibitor), forskolin (a cAMP activator), and vitamin C were used to support cell survival and lineage transition [48].

Under the 6C condition, HTFs gradually transitioned from a flattened fibroblast-like morphology to dome-shaped cells with bright nuclei. Under basal conditions (HTF/iRLC medium without the six-component cocktail), a small fraction of cells (~10%) exhibited *Vsx2*::eGFP positivity, suggesting a low level of spontaneous or primed activation under culture conditions. In contrast, the full 6C cocktail increased the proportion of *Vsx2*::eGFP-positive cells to around 40%, representing a marked enhancement of reporter activation. Consistent with this, automated quantification demonstrated that the complete 6C combination yielded the highest proportion of *Vsx2*::eGFP-positive cells compared with conditions in which individual components were omitted (Figure 2C). Flow cytometric analysis further confirmed induction of *Vsx2*::eGFP-positive cells, with 42.8% of cells falling within the GFP-positive gate under optimized conditions (Figure 2D). These induced retinal lineage–like cells (iRLCs) formed compact clusters after subculture and could be maintained in vitro for more than two months under defined culture conditions (Figure 2E). Time-lapse imaging (images acquired at 30 min intervals) documenting the morphological transition is provided in Videos S1–S4. Time-lapse imaging (day 1–5) further revealed dynamic cellular behaviors during the reprogramming process. Cells exhibited active proliferation during the early phase (day 1 and 2, Video S1; and day 3, Video S2), followed by a marked reduction in cell division after day 4 (Video S3) and day 5 (Video S4). No substantial cell detachment, cell fragmentation, or accumulation of cellular debris was observed during this period, supporting the absence of overt cell death under the present experimental conditions. Karyotype analysis performed before and after reprogramming did not reveal detectable chromosomal abnormalities (Figure S3).

### 3.3. Source-Dependent Reprogramming Responses Among Fibroblast Lines

Previous studies of chemical neuronal conversion have demonstrated that reprogramming efficiency may depend on intrinsic cellular properties as well as environmental factors [51]. To evaluate whether responsiveness to the 6C protocol varied according to tissue origin, we applied identical reprogramming conditions to fibroblasts derived from different anatomical sources, including human fetal lung fibroblasts (IMR-90), neonatal foreskin fibroblasts (CRL-2097 and BJ-5ta), and adult dermal fibroblasts (FB-3652).

**Figure 3:**
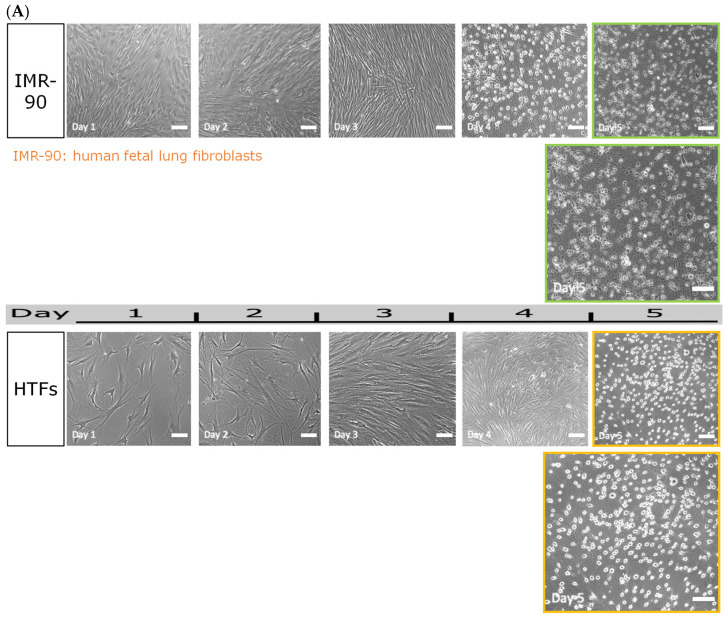

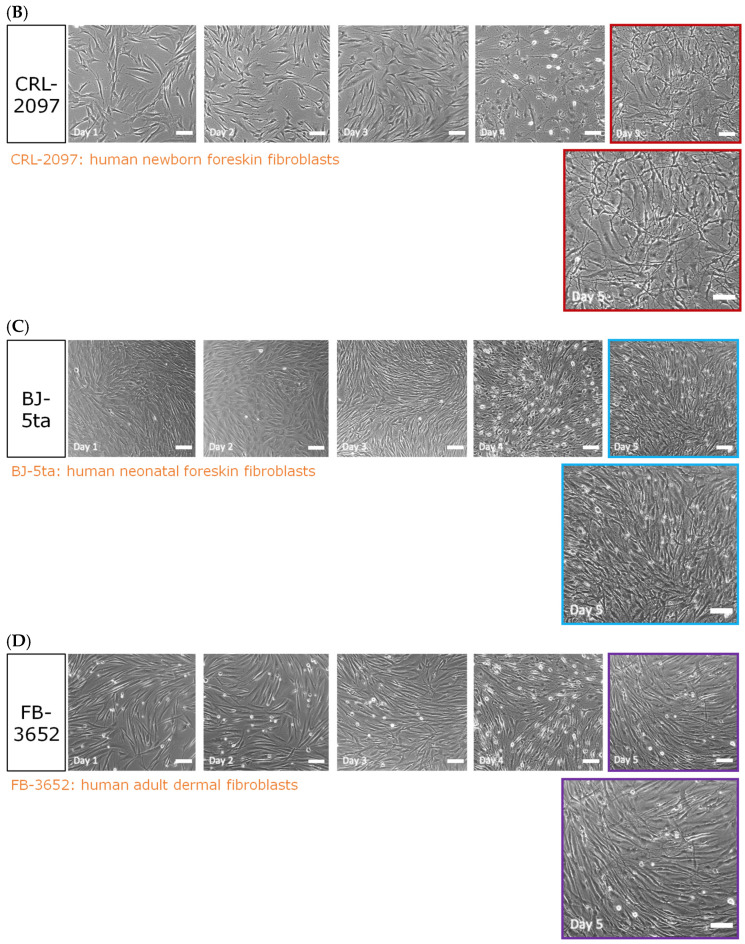
Morphological responses of different fibroblast lines to the 6C reprogramming protocol. Phase-contrast images showing fibroblast lines after application of the 6C protocol. (**A**) HTFs consistently transitioned to dome-shaped cluster-forming cells. IMR-90 cells exhibited reduced viability during treatment. (**B**) CRL-2097 cells adopted elongated morphologies with thin processes. (**C**) BJ-5ta cells retained fibroblast-like morphology. (**D**) FB-3652 cells retained fibroblast-like morphology. Scale bar: 100 μm. Images from HTFs are representative of independent biological replicates derived from different donors (*n* = 6), whereas images from commercial cell lines are representative of independent experiments.

Under these conditions, Tenon’s capsule–derived fibroblasts (HTFs) consistently underwent morphological transition toward dome-shaped, cluster-forming cells. In contrast, other fibroblast lines exhibited limited or variable morphological responses. IMR-90 cells showed reduced viability by day 5 of treatment (Figure 3A). CRL-2097 cells developed elongated morphologies with thin processes (Figure 3B), whereas BJ-5ta and FB-3652 cells largely retained fibroblast-like morphology following exposure to the 6C protocol (Figure 3C,D). These findings indicate differential responsiveness to the 6C condition depending on fibroblast source. Immunofluorescence staining for VSX2 was performed to evaluate lineage-associated induction. VSX2 expression was detected in HTF-derived induced cells but was not observed in non-ocular fibroblast lines under identical conditions (Figure S4). These results support a source-dependent difference in reprogramming responsiveness.

### 3.4. Molecular Characterization of HTF-Derived iRLCs

**Figure 4:**
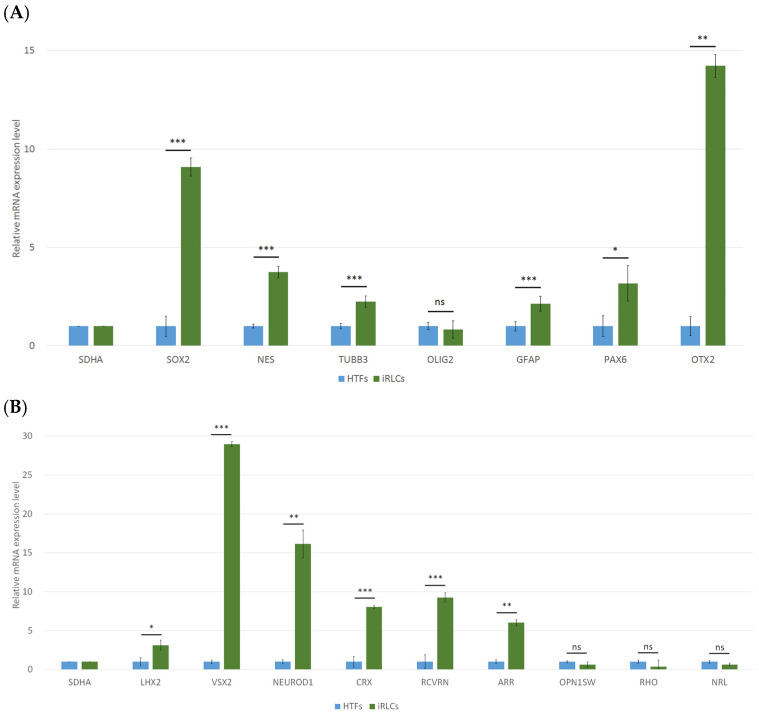

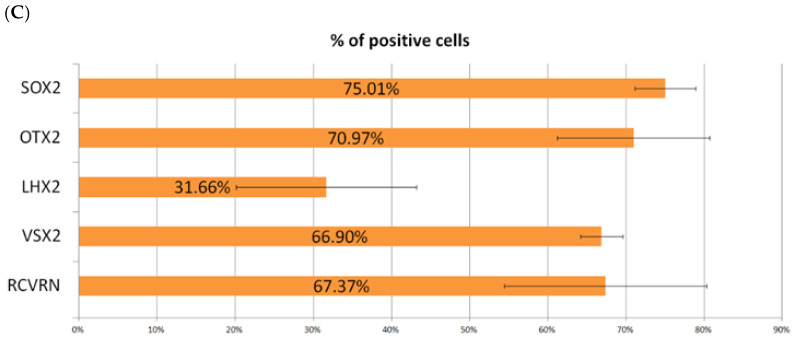
Molecular characterization of iRLCs following chemical reprogramming. (**A**) Quantitative RT-PCR analysis of genes associated with early neural and neuroectodermal programs, including *SOX2*, *NES*, *TUBB3*, *OLIG2*, *GFAP*, *PAX6*, and *OTX2*, in parental HTFs and day 6 iRLCs. (**B**) Quantitative RT-PCR analysis of retinal lineage-associated genes, including *LHX2*, *VSX2*, *NEUROD1*, *CRX*, *RCVRN*, and *ARR*, as well as photoreceptor-associated genes (*OPN1SW*, *RHO*, and *NRL*). Relative expression levels were calculated using the 2^−ΔΔCt^ method and normalized to SDHA. Data are presented as mean ± SD from six independent biological replicates derived from different donors (*n* = 6). Statistical comparisons were performed on ΔCt values using the Mann–Whitney U test with Benjamini–Hochberg FDR correction within each predefined gene panel. ns: not significant; * *p* < 0.05; ** *p* < 0.01; *** *p* < 0.001. (**C**) Quantification of marker-positive cells in iRLC cultures based on immunofluorescence analysis. The proportion of cells expressing each marker was 75.0% for SOX2, 71.0% for OTX2, 31.7% for LHX2, 66.9% for VSX2, and 67.4% for RCVRN (mean ± SD, *n* = 6 independent biological replicates derived from different donors).

To further characterize the molecular identity of HTF-derived induced cells, quantitative RT-PCR was performed on six independent biological replicates derived from different donors (*n* = 6) at day 6 of reprogramming. Relative gene expression levels were calculated using the 2^−ΔΔCt^ method and normalized to SDHA. Fold changes are presented relative to HTFs. Among genes associated with early neural and neuroectodermal programs (Figure 4A), SOX2 expression increased to 9.1 ± 0.5 fold compared with HTFs (*p* < 0.001), and NES increased to 3.8 ± 0.3 fold (*p* < 0.001). TUBB3 and GFAP were elevated to 2.2 ± 0.3 fold and 2.1 ± 0.3 fold, respectively (*p* < 0.001). PAX6 expression increased to 3.2 ± 0.9 fold (*p* < 0.05), and OTX2 increased to 14.4 ± 0.9 fold (*p* < 0.01). OLIG2 did not show a statistically significant change (0.8 ± 0.2 fold, ns). A broader retinal lineage-associated panel was subsequently examined (Figure 4B). LHX2 expression increased to 3.1 ± 0.4 fold (*p* < 0.05), and VSX2 increased to 28.7 ± 1.1 fold (*p* < 0.001). NEUROD1 increased to 16.1 ± 2.3 fold (*p* < 0.01), CRX increased to 8.1 ± 0.7 fold (*p* < 0.001), and RCVRN increased to 9.3 ± 0.7 fold (*p* < 0.001). ARR increased to 6.0 ± 0.5 fold (*p* < 0.01). OPN1SW (0.6 ± 0.2 fold), RHO (0.4 ± 0.2 fold), and NRL (0.6 ± 0.2 fold) did not demonstrate statistically significant changes.

To determine whether mRNA induction corresponded to protein expression, immunofluorescence staining was performed on six independent biological replicates derived from different donors (Figure S5). Quantification of marker-positive cells (Figure 4C) showed that SOX2 was detected in 75.01% of cells, OTX2 in 70.97%, VSX2 in 66.90%, and RCVRN in 67.37%, whereas LHX2 positivity was observed in 31.66% of cells. These findings indicate that a substantial proportion of induced cells express neural and retinal lineage-associated proteins under the reprogramming conditions.

Collectively, the transcriptional and protein-level analyses support activation of neural- and retinal lineage-associated gene programs in HTF-derived iRLCs.

### 3.5. Gene Expression Profile of FACS-Sorted iRPCs

**Figure 5:**
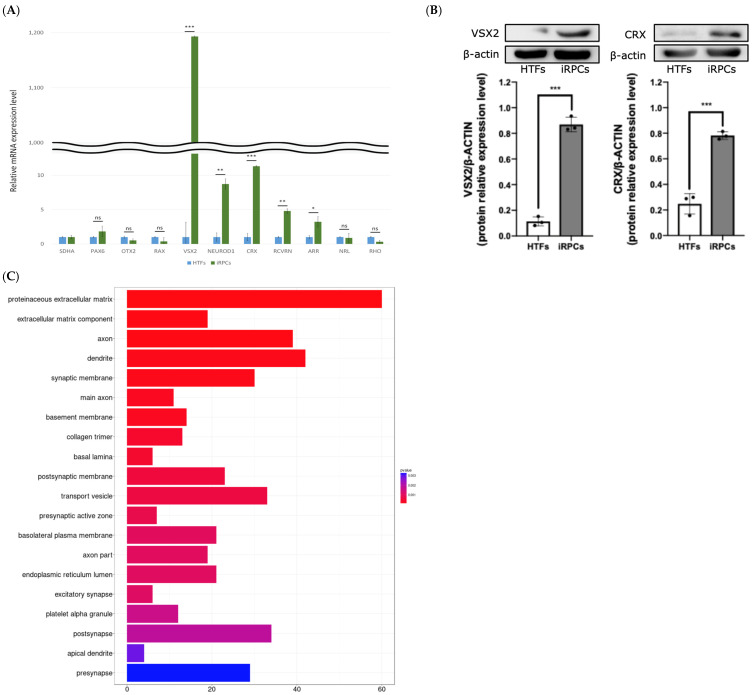

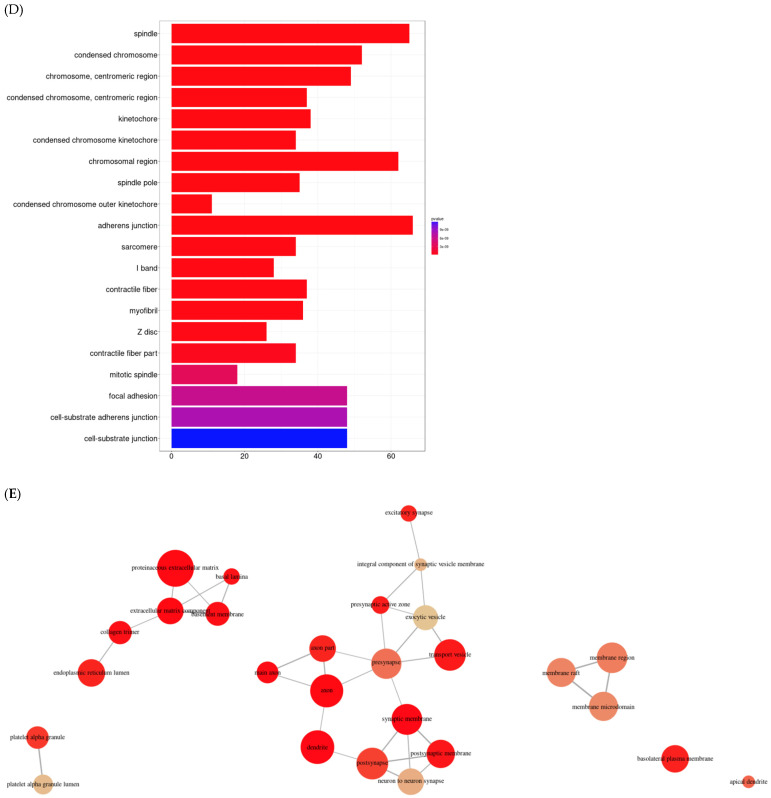
Molecular and transcriptomic characterization of FACS-sorted iRPCs. (**A**) Quantitative RT-PCR analysis of retinal lineage-associated genes (*PAX6*, *OTX2*, *RAX*, *VSX2*, *NEUROD1*, *CRX*, *RCVRN*, *ARR*, *NRL*, and *RHO*) in FACS-isolated iRPCs compared with parental HTFs at day 6 of reprogramming. Relative expression levels were calculated using the 2^−ΔΔCt^ method and normalized to SDHA. Data are presented as mean ± SD from six independent biological replicates derived from different donors (*n* = 6). Statistical analyses were performed on ΔCt values using the Mann–Whitney U test with Benjamini–Hochberg FDR correction. ns: not significant; * *p* < 0.05; ** *p* < 0.01; *** *p* < 0.001. (**B**) Western blot analysis of VSX2 and CRX protein expression in HTFs and iRPCs. Representative blots are shown with β-actin as a loading control. Uncropped full-length Western blot images corresponding to the presented blots are provided in Supplementary Materials (Figure S7). Quantification of band intensity (normalized to β-actin) is presented as mean ± SD from independent biological replicates derived from different donors (*n* = 3). Statistical comparisons were performed using an unpaired two-tailed *t*-test. (**C**) GO enrichment analysis of upregulated genes in iRPCs relative to HTFs, highlighting categories associated with extracellular matrix organization, neuronal projections (axon and dendrite), synaptic membrane components, and vesicle-mediated transport. (**D**) GO enrichment analysis of downregulated genes, including categories related to mitotic processes, chromosomal organization, spindle assembly, and cell cycle-associated structures. (**E**) Enrichment map of KEGG pathways derived from differentially expressed genes. Clusters represent functionally related pathway groups, including extracellular matrix-associated processes and neuronal/synaptic signaling modules. Node size reflects pathway connectivity, and edges indicate shared genes between pathways.

To further evaluate the transcriptional characteristics of the enriched population, quantitative RT-PCR was performed on six independent biological replicates derived from different donors, FACS-sorted iRPCs compared with parental HTFs (Figure 5A). Relative expression levels were calculated using the 2^−ΔΔCt^ method and normalized to SDHA. VSX2 expression increased to 1190-fold relative to HTFs (*p* < 0.001), representing the most prominent transcriptional change observed in the sorted population. NEUROD1, CRX, RCVRN, and ARR were also significantly upregulated (*p* < 0.05 or *p* < 0.01 or *p* < 0.001, as indicated). In contrast, PAX6, OTX2, RAX, NRL, and RHO did not demonstrate statistically significant differences under these conditions. These findings indicate enrichment of retinal lineage-associated gene expression within the FACS-sorted iRPC fraction.

To further validate these transcriptional changes at the protein level, Western blot analysis was performed for selected retinal lineage markers. Consistent with the qRT-PCR results, VSX2 and CRX protein expression levels were increased in FACS-sorted iRPCs compared with parental HTFs (Figure 5B). Densitometric analysis normalized to β-actin demonstrated a significant upregulation of both proteins in iRPCs (*n* = 3). These findings support the acquisition of retinal lineage-associated molecular features at both the transcriptional and protein levels.

To characterize global transcriptional alterations, bulk RNA sequencing was performed on HTFs and iRPCs. Gene Ontology (GO) enrichment analysis identified significantly enriched categories among upregulated genes related to extracellular matrix components, axonal and dendritic structures, synaptic membrane regions, and vesicle-associated processes (Figure 5C). Downregulated GO terms were enriched for categories associated with spindle organization, chromosomal structures, and contractile or adhesion-related components (Figure 5D). Kyoto Encyclopedia of Genes and Genomes (KEGG) pathway enrichment analysis similarly demonstrated clusters associated with synaptic membrane regions and extracellular matrix–related pathways (Figure 5E). Category network (cnet) analysis illustrated clustering of upregulated genes within structural and membrane-associated functional groups (Figure S6). Consistent with the observed reduction in proliferative activity, RNA sequencing analysis revealed downregulation of proliferation- and cell cycle-associated genes in iRPCs compared to HTFs, including MKI67, MCM2, MCM5, MCM7, TOP2A, and CCNB1 (Table S5). These findings further support a transition toward a less proliferative and more lineage-committed state during chemical reprogramming.

Collectively, transcriptomic profiling indicates substantial remodeling of gene expression programs in iRPCs compared with parental HTFs.

### 3.6. In Vitro Calcium Imaging

While gene expression profiles provide insight into cellular identity, functional behavior should be interpreted in conjunction with molecular characteristics to more comprehensively evaluate cellular properties [52]. In the retina, the majority of excitatory neuronal populations—including photoreceptors and bipolar cells—utilize glutamate as their primary neurotransmitter. Previous studies have also reported that fetal RPCs exhibit intracellular calcium responses upon stimulation with 1 mM glutamate [18].

To examine whether chemically reprogrammed cells display comparable glutamate responsiveness, intracellular calcium dynamics were assessed using Fura-2–based live-cell imaging. Cells were loaded with Fura-2 AM and stimulated with 1 mM glutamate, and fluorescence intensity was continuously recorded. Glutamate was applied between 100 and 400 s during acquisition, and changes in fluorescence were analyzed as indicators of intracellular calcium influx.

**Figure 6:**
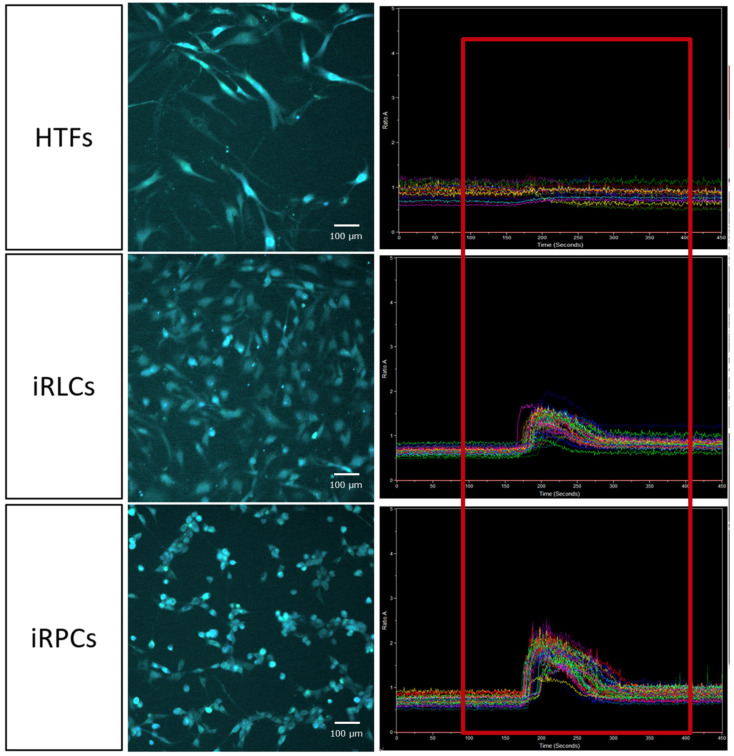
Glutamate-evoked intracellular calcium responses in HTFs, iRLCs, and FACS-sorted iRPCs. Representative Fura-2 fluorescence images (**left**) and corresponding intracellular calcium traces (**right**) recorded following stimulation with 1 mM glutamate. Glutamate was applied between 100 and 400 s (red box). HTFs showed no detectable changes in intracellular calcium levels during stimulation. In contrast, iRLCs exhibited reproducible increases in intracellular calcium levels upon glutamate exposure, and FACS-sorted iRPCs demonstrated more prominent calcium transients under identical conditions. Scale bar, 100 μm. Images and traces are representative of independent biological replicates derived from different donors (*n* = 6).

Parental HTFs did not exhibit detectable changes in intracellular calcium levels upon glutamate stimulation, and calcium traces remained near baseline throughout the recording period. In contrast, iRLCs demonstrated reproducible increases in intracellular calcium levels during glutamate exposure. FACS-sorted iRPCs exhibited more pronounced calcium transients compared with unsorted iRLCs under identical stimulation conditions (Figure 6).

These observations indicate that chemically reprogrammed cells display glutamate-responsive calcium signaling activity in vitro, whereas parental fibroblasts do not demonstrate comparable responsiveness under the same experimental conditions.

### 3.7. Intravitreal Transplantation in Healthy Wistar Rats

To evaluate short-term ocular tolerability following intravitreal delivery, PBS, HTFs, or unsorted iRLCs were injected into the vitreous cavity of adult albino Wistar rats (*n* = 6 animals per group) as described in Section 2.11. Structural and functional assessments were performed one month after transplantation.

**Figure 7:**
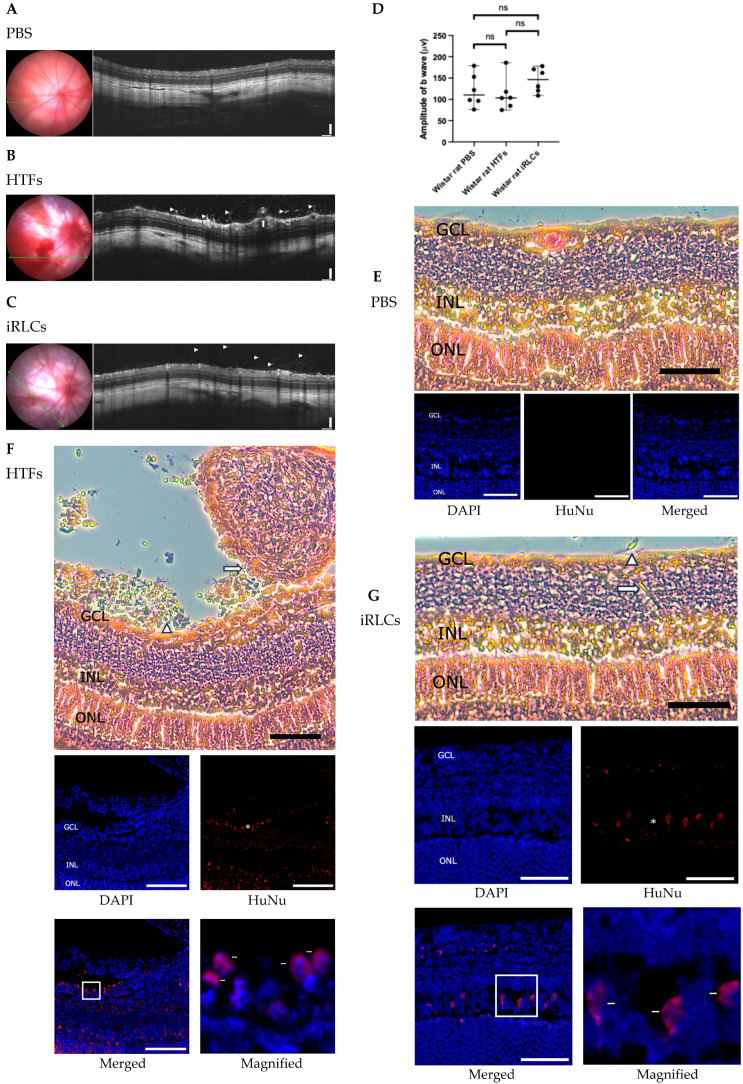
Structural and histological assessment one month after intravitreal transplantation in healthy Wistar rats. (**A**) Representative fundus photographs and OCT images of PBS-treated eyes showing recognizable retinal layer organization without evident vitreoretinal surface abnormalities one month after injection. (**B**) HTF-transplanted eyes showing hyperreflective material in the vitreous (triangles) and irregularity of the vitreoretinal surface (arrow), consistent with epiretinal membrane–like changes and localized structural alterations. (**C**) iRLC-transplanted eyes exhibiting accumulation of hyperreflective material along the retinal surface (triangles), without overt disruption of underlying retinal layers under the present experimental conditions. (**D**) Scotopic ERG recordings obtained one month after transplantation. Quantification of b-wave amplitudes showed no statistically significant differences among PBS-, HTF-, and iRLC-treated eyes (mean ± SD, *n* = 6 animals per group; ns, not significant). (**E**) Representative H&E staining of retinal sections from PBS-treated eyes showing recognizable retinal layering, including GCL, INL, and ONL. (**F**) HTF-transplanted eyes showing formation of fibrotic membrane–like structures along the retinal surface. A dense epiretinal cell mass (arrow) and associated localized alterations in retinal organization (triangle) were observed. Immunofluorescence staining revealed HuNu-positive signals (asterisk) within the epiretinal membrane. HuNu-positive signals were identified based on nuclear morphology and co-localization with DAPI in magnified views (arrow). (**G**) In iRLC-transplanted eyes, retinal layer organization was largely discernible, and a limited number of cells with dome-shaped morphology and bright nuclei were observed on the retinal surface (triangle) and within retinal layers (arrow). HuNu-positive signals (asterisk) were identified based on nuclear morphology and co-localization with DAPI in magnified views (arrow). Scale bars: 50 μm. All representative fluorescence images were acquired using confocal microscopy (Leica TCS SP8) to ensure optical sectioning and accurate nuclear localization. Images are representative of all animals analyzed (*n* = 6 per group).

OCT imaging showed recognizable retinal layer organization in PBS-treated eyes without evident vitreoretinal surface abnormalities (Figure 7A). In contrast, HTF-transplanted eyes exhibited hyperreflective epiretinal membrane–like structures and surface irregularities along the vitreoretinal interface (Figure 7B). Eyes receiving iRLCs displayed accumulation of hyperreflective material along the retinal surface without obvious disruption of retinal laminar architecture under the present experimental conditions (Figure 7C). These OCT findings should be interpreted as indirect structural features rather than direct visualization of transplanted cells, given the resolution limitations of OCT imaging under the present experimental conditions. Scotopic ERG recordings obtained one month after transplantation did not reveal statistically significant differences in b-wave amplitudes among PBS-, HTF-, and iRLC-treated eyes under the present experimental conditions (Figure 7D).

Histological analysis further supported these observations. H&E staining of PBS-treated eyes demonstrated recognizable retinal layering, including the ganglion cell layer (GCL), inner nuclear layer (INL), and outer nuclear layer (ONL) (Figure 7E). In HTF-transplanted eyes, H&E staining revealed fibrotic membrane–like structures adherent to the retinal surface, accompanied by localized alterations in retinal organization (Figure 7F). In iRLC-transplanted eyes, retinal lamination remained largely discernible, with identifiable GCL, INL, and ONL, although mild and localized irregularities were occasionally observed (Figure 7G). Importantly, these changes were focal and did not preclude evaluation of retinal layer organization. Immunofluorescence analysis using a human-specific nuclear marker (HuNu) revealed detectable HuNu-positive signals within these epiretinal structures in HTF-transplanted eyes. In contrast, iRLC-transplanted eyes exhibited generally maintained retinal layer organization, without obvious disruption of laminar architecture. HuNu-positive signals were detected in a subset of sections, including regions corresponding to the GCL and INL, and occasionally along the retinal surface (Figure 7G). A semi-quantitative assessment was performed to evaluate the distribution of transplanted cells. Rather than enumerating absolute cell numbers, we assessed the proportion of eyes with detectable HuNu-positive signals and the overall distribution patterns across sections. HuNu-positive signals were consistently observed in HTF-transplanted eyes, predominantly associated with epiretinal membrane–like structures, whereas in iRLC-transplanted eyes, HuNu-positive signals were detected in a subset of animals (4 out of 6), exhibiting a more localized and heterogeneous distribution. Additional representative sections from independent animals further illustrated similar distribution patterns across groups, with minimal HuNu-positive signal in PBS-treated eyes, prominent epiretinal accumulation in HTF-transplanted eyes, and sparse, localized HuNu-positive signals in iRLC-transplanted eyes (Figure S8). Given the localized and non-uniform distribution of transplanted cells within the spherical ocular environment, these findings are interpreted as indicating limited persistence and spatially restricted localization under the present experimental conditions, rather than uniform engraftment or widespread integration.

### 3.8. Subretinal Transplantation in Healthy Long–Evans Rats

To further assess retinal tolerability following subretinal delivery, healthy adult Long–Evans rats received subretinal injections of PBS, HTFs, or unsorted iRLCs (*n* = 6 animals per group) as described in Section 2.12. Structural and functional assessments were performed one month after transplantation.

**Figure 8:**
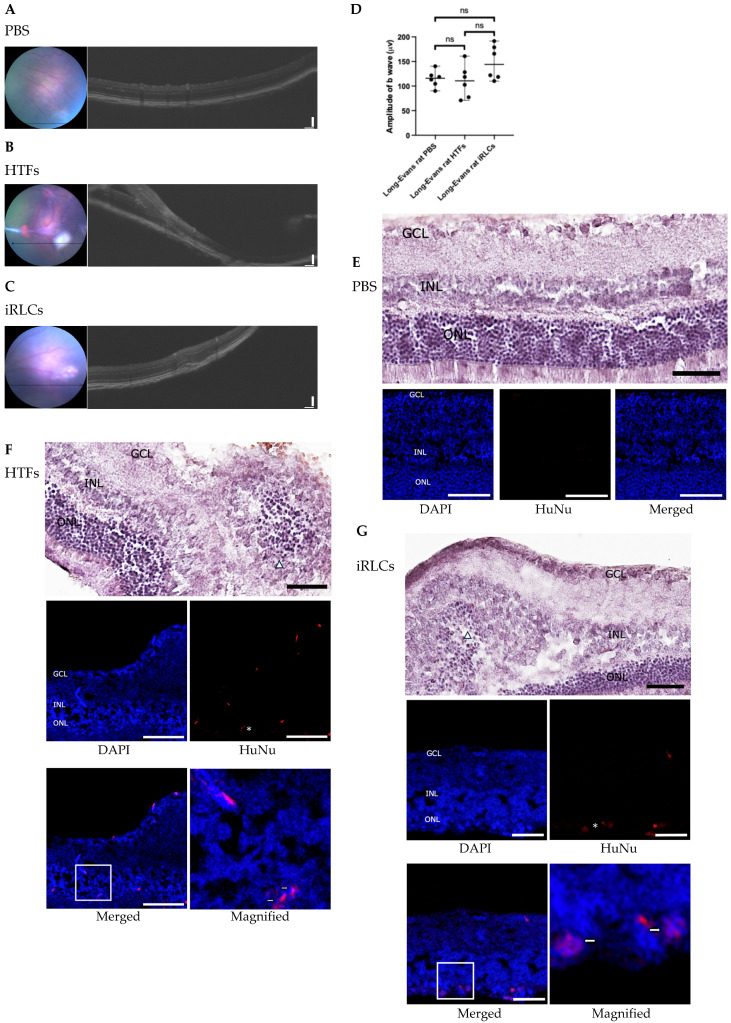
Structural and histological assessment one month after subretinal transplantation in healthy Long–Evans rats. (**A**) Representative fundus photographs and OCT images of PBS-treated eyes showing recognizable retinal layer organization at the injection site one month after surgery without evident structural abnormalities. (**B**) HTF-transplanted eyes showing epiretinal cell accumulation on fundus imaging and associated retinal surface irregularity and traction on OCT. (**C**) iRLC-transplanted eyes exhibiting generally maintained retinal layer organization, with localized subretinal elevation corresponding to the injection site, consistent with subretinal bleb formation under the present experimental conditions. (**D**) Scotopic ERG recordings obtained one month after transplantation. Quantification of b-wave amplitudes showed no statistically significant differences among PBS-, HTF-, and iRLC-treated eyes (mean ± SD, *n* = 6 animals per group; ns, not significant). (**E**) Representative H&E staining of retinal sections from PBS-treated eyes demonstrating recognizable retinal layering, including GCL, INL, and ONL. (**F**) HTF-transplanted eyes showing fibrotic membrane formation along the retinal surface, accompanied by retinal traction and localized alterations in retinal organization (triangle). Cells within the epiretinal membrane showed detectable HuNu-positive signals (asterisk), with co-localization with DAPI in magnified views (arrow). (**G**) iRLC-transplanted eyes exhibiting localized alterations in retinal layer organization at the injection site (triangle), including focal intermixing of the INL and ONL. HuNu-positive signals (asterisk) were identified based on nuclear morphology and co-localization with DAPI in magnified views (arrow). These signals were sparse and heterogeneously distributed, and are therefore conservatively interpreted as indicative of limited short-term cellular persistence. Scale bars: 50 μm. Images are representative of all animals analyzed (*n* = 6 per group).

OCT imaging showed recognizable retinal layer organization in PBS-treated eyes without evident structural abnormalities at the injection site (Figure 8A). HTF-transplanted eyes exhibited epiretinal membrane formation, retinal traction, and localized structural irregularities (Figure 8B). In contrast, iRLC-transplanted eyes displayed localized subretinal elevation corresponding to the injection site, consistent with subretinal bleb formation following injection, without evidence of widespread retinal abnormalities under the present experimental conditions (Figure 8C). Scotopic ERG recordings obtained one month after transplantation did not reveal statistically significant differences in b-wave amplitudes among PBS-, HTF-, and iRLC-treated eyes (Figure 8D), suggesting no detectable functional impairment under the present experimental conditions.

Histological analysis further supported these observations. H&E staining of PBS-treated eyes demonstrated recognizable retinal layering, including the GCL, INL, and ONL (Figure 8E). In HTF-transplanted eyes, fibrotic membrane–like structures were observed along the retinal surface, accompanied by retinal traction and localized alterations in retinal organization (Figure 8F). Immunofluorescence analysis revealed detectable HuNu-positive signals within these epiretinal structures. In contrast, iRLC-transplanted eyes exhibited localized structural alterations at the injection site, including focal alterations in retinal laminar organization and partial intermixing of the INL and ONL, without evidence of widespread retinal abnormalities (Figure 8G). These changes were spatially restricted and are consistent with localized mechanical effects associated with subretinal injection, where transient separation of the neural retina from the underlying layers is expected. These localized changes were not observed in PBS-treated eyes. HuNu-positive signals were detected in a subset of sections, based on nuclear morphology and co-localization with DAPI. These signals were spatially localized and exhibited a heterogeneous distribution pattern. A distribution-based semi-quantitative assessment was performed to evaluate the spatial pattern of transplanted cells. Rather than enumerating absolute cell numbers, we assessed the proportion of eyes with detectable HuNu-positive signals and the overall distribution patterns across sections. HuNu-positive signals were consistently observed in HTF-transplanted eyes, predominantly associated with fibrotic membrane–like structures, whereas in iRLC-transplanted eyes, HuNu-positive signals were detected in 4 out of 6 animals, with signals primarily observed in regions corresponding to the subretinal space. Additional representative sections from independent animals further illustrated similar distribution patterns across groups (Figure S9). Given the localized distribution of transplanted cells within the subretinal space and localized alteration in retinal layer organization, these findings are conservatively interpreted as indicating limited persistence and spatially restricted localization under the present experimental conditions. These observations should therefore be considered preliminary, and further studies with extended follow-up, improved detection strategies, and quantitative analyses will be necessary to clarify the persistence, distribution, and biological impact of transplanted cells.

## 4. Discussion

### 4.1. Insights

This study explored a small-molecule–based direct reprogramming strategy to shift adult human Tenon’s capsule fibroblasts (HTFs) toward a retinal progenitor–like state without exogenous transcription factor delivery. Compared with iPSC/ESC-based approaches that proceed through pluripotency and staged differentiation, direct chemical reprogramming leverages defined compounds to modulate epigenetic state, cell cycle regulation, and lineage-associated transcriptional programs. In the present protocol, RG108 and VPA were applied during the early phase, followed by SU9516 and a survival/supportive phase including Y-27632, forskolin, and vitamin C (Figure 2), consistent with prior chemical reprogramming logic and reported compound functions.

A key practical consideration in direct reprogramming is the choice of starting somatic cell source. Multiple tissue sources—including dermal fibroblasts and other accessible somatic cells—have been investigated across reprogramming studies [53], and intrinsic reprogramming competence has been reported to vary by anatomical origin in neuronal conversion paradigms [51]. In this study, Tenon’s capsule tissue reproducibly generated adherent fibroblast-like cultures, whereas vitreous samples did not yield proliferative adherent cells under the conditions tested (Figure 1A). Flow cytometry further supported a fibroblast identity for Tenon’s capsule–derived cultures based on high S100A4 positivity (Figure 1B). When the 6C condition was applied across fibroblast lines from different anatomical sources, HTFs exhibited a reproducible morphological transition toward dome-shaped, cluster-forming cells, whereas non-ocular fibroblast lines showed limited or distinct responses and lacked detectable VSX2 induction by immunofluorescence under identical conditions (Figure 3 and Figure S4). Together, these observations support source-dependent responsiveness to the 6C protocol.

At the molecular level, day 6 iRLCs demonstrated coordinated induction of neural/neuroectodermal and retinal lineage-associated transcripts relative to parental HTFs, including VSX2 and additional retinal development markers (Figure 4A,B). Protein-level immunofluorescence further showed that a substantial fraction of iRLCs expressed SOX2, OTX2, VSX2, and RCVRN, while LHX2 positivity was lower, consistent with cellular heterogeneity within the induced population (Figure 4C). This heterogeneity is also consistent with the observation that only a subset of reporter cells became *Vsx2*::eGFP-positive during optimization (Figure 2D). FACS enrichment provided an operational approach to define and characterize a VSX2-enriched fraction (iRPCs), in which VSX2 induction was markedly increased by qRT-PCR (Figure 5A). Consistent with these findings, Western blot analysis further supported the upregulation of retinal lineage-associated proteins, including VSX2 and CRX, in iRPCs compared with parental HTFs (Figure 5B). Importantly, these fold-change values reflect induction from negligible baseline expression in fibroblasts, and should be interpreted as evidence of directional transcriptional remodeling rather than equivalence to bona fide human RPCs.

Transcriptomic profiling by bulk RNA sequencing and enrichment analyses indicated broad gene program remodeling in iRPCs relative to HTFs, with enriched categories related to extracellular matrix, neuronal/synaptic-associated terms, and reduced enrichment of mitotic/spindle-related categories (Figure 5C,D). These analyses are best interpreted as exploratory and hypothesis-generating, particularly given the use of bulk profiling and enrichment-based inference.

Functional assays were used to complement marker-based characterization. Because retinal neuronal circuits include glutamatergic signaling, and fetal RPCs have been reported to exhibit intracellular calcium responses to 1 mM glutamate [18], glutamate-evoked Ca^2+^ imaging was applied as an in vitro physiological readout. Under the present conditions, parental HTFs did not show detectable glutamate-evoked calcium transients, whereas iRLCs and FACS-sorted iRPCs exhibited stimulus-associated calcium influxes (Figure 6). These data suggest acquisition of neural-responsive properties in induced cells, although they do not establish functional equivalence to native retinal progenitors or mature retinal neurons [52].

For in vivo feasibility and short-term tolerability, induced cells were delivered by intravitreal injection in Wistar rats and subretinal injection in Long–Evans rats. These two ocular delivery routes have also been adopted in clinical-stage studies of human RPC products, including intravitreal administration in the jCyte program [36] and subretinal transplantation in the ReNeuron program [35], thereby providing a translational context for evaluating route-dependent tissue responses. Across both paradigms, OCT and histological analyses showed structural alterations and fibrotic membrane–like changes in eyes receiving parental fibroblasts. iRLC-treated eyes exhibited localized alterations in retinal layer organization at the subretinal injection site, including focal alterations in laminar architecture and partial intermixing of the INL and ONL, whereas PBS-treated eyes showed recognizable retinal layering under the present experimental conditions. These changes were spatially restricted and not associated with detectable differences in scotopic ERG responses at the one-month time point. Human nuclei-positive signals were detectable in a subset of sections, exhibiting a focal and heterogeneous distribution pattern across examined eyes (Figure 7 and Figure 8). In contrast, scotopic ERG recordings did not show statistically significant differences among PBS-, HTF-, and iRLC-treated groups under the present experimental conditions (Figure 7D and Figure 8D). Collectively, these findings suggest a differential structural response between fibroblast- and iRLC-treated eyes, with localized structural alterations observed following iRLC delivery but without detectable functional impairment at the one-month time point. Importantly, these findings should be interpreted as preliminary and descriptive, reflecting short-term cell presence and delivery feasibility rather than evidence of widespread engraftment or functional integration.

### 4.2. Limitations

The present study has several limitations that should be considered when interpreting the findings.

First, the induced cell populations exhibited heterogeneity in reporter activation and lineage-associated marker expression. Although FACS enrichment enabled characterization of a VSX2-enriched fraction, the study does not establish uniform lineage conversion or define cell-state composition at single-cell resolution. More detailed analyses using single-cell transcriptomic or multi-marker co-expression approaches will be required to more precisely characterize population heterogeneity.

Second, interpretation of fold-change–based gene expression requires caution. Many retinal lineage genes exhibit low baseline expression levels in fibroblasts; therefore, large relative fold inductions (e.g., VSX2) reflect substantial transcriptional upregulation relative to the starting population rather than direct equivalence to native RPCs. Consistent with this, not all retinal maturation markers were robustly induced in iRLCs, and the induced cells are best interpreted as progenitor-like or partially lineage-shifted states rather than terminally differentiated retinal cell types.

Third, functional characterization was limited in scope. Glutamate-evoked Ca^2+^ imaging was performed using a single stimulant concentration (1 mM) based on prior fetal retinal progenitor observations [18] and served as a complementary functional readout to molecular profiling. However, receptor subtype specificity, pharmacological blockade, and dose–response relationships were not investigated. Accordingly, the observed Ca^2+^ responses indicate stimulus-associated calcium dynamics but do not establish physiological equivalence to native retinal neurons or progenitors [52].

Fourth, transcriptomic inference from bulk RNA sequencing remains exploratory. Enrichment analyses identify overrepresented functional categories but cannot resolve cellular heterogeneity, direct regulatory mechanisms, or lineage trajectory at single-cell resolution. Therefore, pathway-level interpretations should be considered hypothesis-generating.

Fifth, the in vivo experiments provide short-term structural and tolerability observations rather than definitive safety or efficacy conclusions. Although OCT and histological analyses demonstrated differential structural responses between fibroblast- and iRLC-treated eyes, scotopic ERG recordings did not show statistically significant differences among treatment groups at the one-month endpoint. Thus, functional interpretation should remain conservative. In addition, histological analysis was based on selected sections from each eye, which represent only partial sampling of a three-dimensional structure. Therefore, detection of transplanted cells may be influenced by section selection and sampling variability. In particular, localized alterations in retinal laminar organization observed following subretinal delivery should be interpreted cautiously and require validation in larger cohorts and longer-term studies. The current animal studies were not designed as GLP-compliant toxicology assessments, and additional safety endpoints—including inflammatory, proliferative, and apoptosis markers—together with longer-term follow-up will be necessary.

Finally, while human nuclei-positive signals were detectable within retinal layers following transplantation, the present study does not establish donor-derived retinal differentiation, synaptic integration, or durable functional contribution. Future investigations incorporating higher-resolution lineage tracing, integration assays, and retina-specific functional testing will be required to clarify long-term biological impact.

## 5. Conclusions

In conclusion, this study demonstrates that human Tenon’s capsule–derived fibroblasts can undergo small-molecule–mediated transcriptional remodeling toward a retinal progenitor–like state under defined culture conditions. Induced cells exhibited neural- and retinal lineage-associated molecular features and glutamate-responsive calcium dynamics in vitro.

Following intravitreal and subretinal delivery, human nuclei-positive signals were detectable in a subset of eyes, exhibiting focal and heterogeneous distribution patterns. While localized alterations in retinal structure following subretinal delivery were noted, no detectable changes in scotopic ERG responses were observed at the one-month time point. These findings suggest short-term functional tolerability and limited persistence, rather than evidence of widespread engraftment or functional integration.

These results provide preliminary feasibility-level evidence supporting further investigation of chemically induced retinal progenitor–like cells as an exploratory cellular platform. Future studies incorporating larger cohorts, retina-specific functional assays, extended follow-up, and higher-resolution analyses will be required to further define biological properties and translational relevance.

## 6. Patents

PCT and TW patent applications were filed by Academia Sinica and Hualien Tzu Chi Hospital; the status of the applications is pending (ACA0164PCT, ACA0164TW).

## Data Availability

Bulk RNA-sequencing data has been released in GEO database (Accession Number: GSE297877).

## Supplementary Materials

The following supporting information can be downloaded at: https://www.mdpi.com/article/10.3390/jfb17050236/s1, Figure S1. Validation of lentiviral transduction efficiency in HTFs using eGFP reporter control; Figure S2. (A) Library preparation and customized bioinformatics workflow; Figure S3. Karyotyping of HTFs and iRLCs; Figure S4. VSX2 expression following reprogramming in different fibroblast sources; Figure S5. Immunofluorescence analysis of neural and retinal lineage-associated protein expression in HTFs and iRLCs; Figure S6. Cnet plot illustrating representative upregulated genes contributing to enriched biological process and cellular component categories; Figure S7. Uncropped Western blot images corresponding to Figure 5B; Figure S8. Additional representative immunofluorescence images from independent animals following intravitreal transplantation; Figure S9. Additional representative immunofluorescence images from independent animals following subretinal transplantation; Table S1. Summary of studies using human retinal progenitor cells on retinal diseases; Table S2: *Vsx2*::eGFP promoter reporter sequences; Table S3: qRT-PCR primer sequences; Table S4. Summary of studies on transplantation of RPCs in RCS rats; Table S5. Proliferation- and cell cycle-associated genes in RNA sequencing; Video S1. Time-lapse video of cell morphology on day 1 and 2 of reprogramming; Video S2. Time-lapse video of cell morphology on day 3 of reprogramming; Video S3. Time-lapse video of cell morphology on day 4 of reprogramming; Video S4. Time-lapse video of cell morphology on day 5 of reprogramming.

## Abbreviations

The following abbreviations are used in this manuscript:

RP	retinitis pigmentosa
DR	diabetic retinopathy
AMD	age-related macular degeneration
DM	diabetes mellitus
RPE	retinal pigment epithelium
ESCs	embryonic stem cells
iPSCs	induced pluripotent stem cells
RPCs	retinal progenitor cells
HTFs	Human Tenon’s capsule fibroblasts
FBS	fetal bovine serum
PBS	phosphate-buffered saline
BSA	bovine serum albumin
MOI	multiplicity of infection
iRLCs	induced retinal lineage-like cells
ISCN	International System for Human Cytogenetic Nomenclature
FACS	fluorescence-activated cell sorting
qRT-PCR	Real-time quantitative reverse-transcription PCR
PBST	Phosphate-buffered saline with Tween 20
HRP	Horseradish peroxidase
RIN	RNA integrity number
PE	paired-end
BSS	balanced salt solution
OCT	optical coherence tomography
ERG	electroretinogram
ANOVA	analysis of variance
FDR	false discovery rate
S100A4	S100 calcium-binding protein A4
6C	six-compound
VPA	valproic acid
FSK	forskolin
VC	vitamin C
DNMT	DNA methyltransferase
GO	Gene Ontology
Cnet	category network
KEGG	Kyoto Encyclopedia of Genes and Genomes
INL	inner nuclear layer
GCL	ganglion cell layer
ONL	outer nuclear layer
