# Supplementary material for: Direct Chemical Reprogramming of Human Fibroblasts into Retinal Progenitor-like Cells for Ocular Delivery
Source: J Funct Biomater. 2026 May 8;17(5):236. doi: 10.3390/jfb17050236 (PMC13208236; doi:10.3390/jfb17050236)
Supplement: Supplementary file 1 [file jfb-17-00236-s001.zip › Figure S1.pdf]

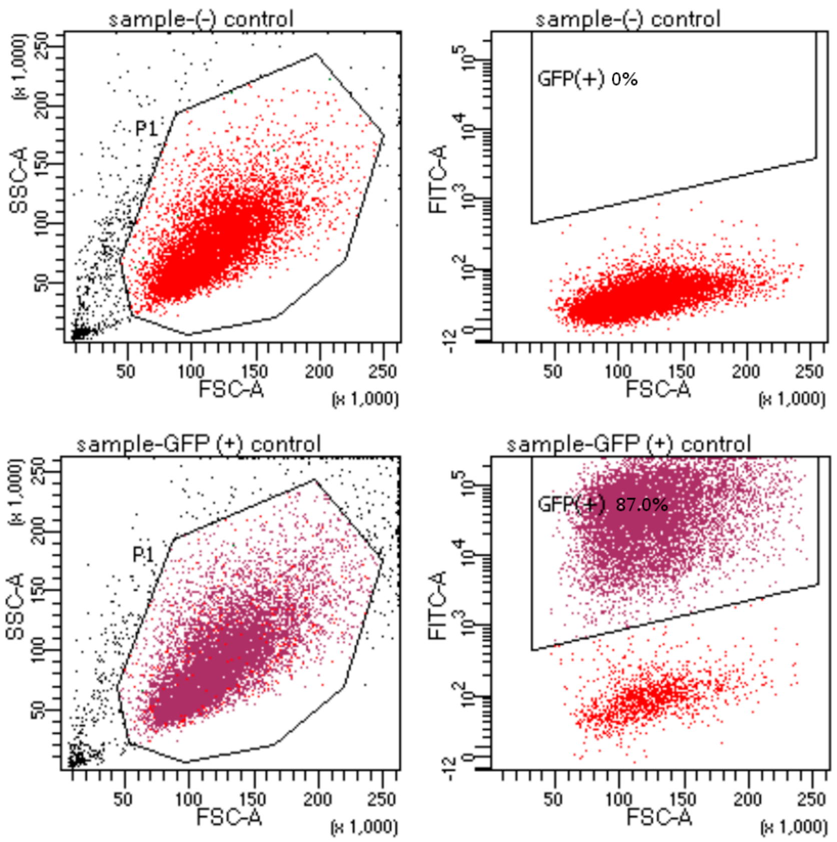


**Figure S1. Figure S1. Validation of lentiviral transduction efficiency in HTFs using eGFP reporter control.** Representative flow cytometry plots demonstrating transduction efficiency of HTFs with the control lentiviral construct pLKO_AS7w.eGFP.puro. Upper panels: sample (−) control showing FSC-A/SSC-A gating (P1) and background FITC signal. The GFP-positive gate was defined based on the fluorescence distribution of the negative control. Lower panels: HTFs transduced at MOI 20 with pLKO_AS7w.eGFP.puro. Within the P1 population, 87.0% of cells fell within the GFP-positive gate. Data are representative of six independent transductions. FSC-A, forward scatter area; SSC-A, side scatter area; FITC-A, fluorescein isothiocyanate fluorescence channel used to detect eGFP signal.
