# Supplementary material for: Direct Chemical Reprogramming of Human Fibroblasts into Retinal Progenitor-like Cells for Ocular Delivery
Source: J Funct Biomater. 2026 May 8;17(5):236. doi: 10.3390/jfb17050236 (PMC13208236; doi:10.3390/jfb17050236)
Supplement: Supplementary file 1 [file jfb-17-00236-s001.zip › Figure S2.pdf]

A

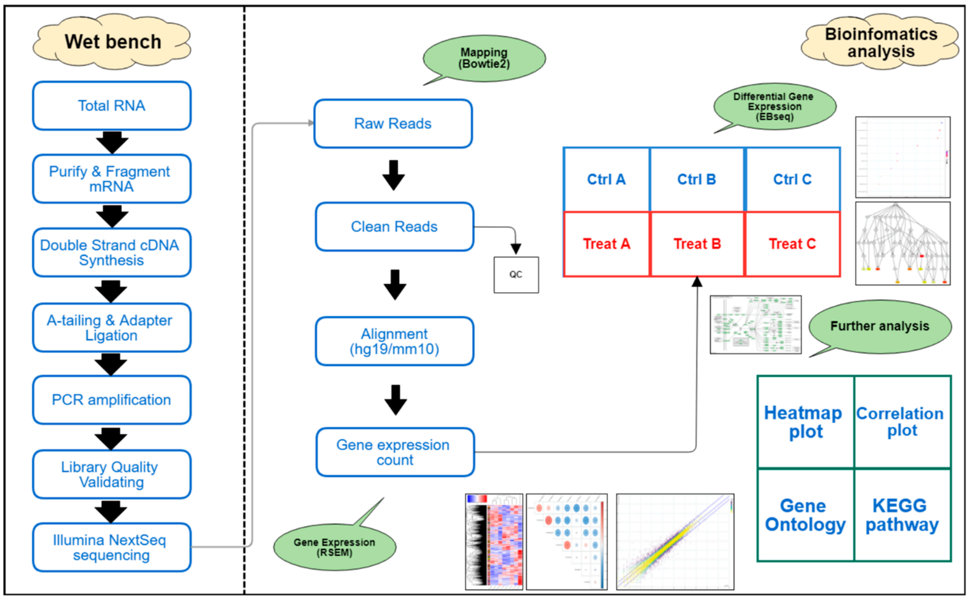

B

Sample_Name	Alignment_Rate	Condition	Output_Name	Sample_Project
HTF4	84.85%	HTF4	HTF4	HS21735
iRPC4	85.75%	iRPC4	iRPC4	HS21735

C

Sample_Name	File_Name	GC.	Length	Length_Mean	Phred	Q20_Ratio	Q30_Ratio	Qual_Mean	Read_Counts	Total_Bases
HTF4_R1	HTF4_R1.clean.fastq.gz	50.20%	20-151	144	33	98.75%	95.54%	36.29	24,820,100	3,574,131,281
HTF4_R2	HTF4_R2.clean.fastq.gz	50.30%	20-151	143.1	33	98.06%	93.58%	35.96	24,820,100	3,551,633,694
iRPC4_R1	iRPC4_R1.clean.fastq.gz	49.95%	20-151	143.38	33	98.74%	95.54%	36.29	32,041,814	4,594,080,180
iRPC4_R2	iRPC4_R2.clean.fastq.gz	50.08%	20-151	142.75	33	98.30%	94.23%	36.07	32,041,814	4,574,032,861

Figure S2. A. Library preparation and customized bioinformatics workflow.

Purify and fragment mRNA: Using poly-T oligo-attached beads to purify mRNA, which is also fragmented primed for cDNA synthesis. First and second strand cDNA synthesis: Using reverse transcriptase and random primer to synthesize first strand cDNA, and using dUTP in place of dTTP to generate double-strand cDNA. A-tailing and Adaptor Ligation: A single ‘A’ nucleotide is added to 3’ end of ds cDNAs. Then, multiple indexing adapters are ligated to 5’ and 3’ of the ends of the ds cDNA. PCR amplification: Using PCR to selectively amplify those DNA fragments that have

adapters on both ends. Library quality validating: Library was validated on Agilent 2100 Bio-analyzer and Real-Time PCR System. **B. RNA-seq alignment summary for representative samples.** Alignment rate for raw RNA-seq reads from human Tenon's fibroblasts (HTF4) is 84.85%, and FACS-sorted induced retinal progenitor–like cells (iRPC4) is 85.75%. **C. RNA-seq sequencing quality metrics for HTF4 and iRPC4 samples.** Metrics include GC content, read length distribution, mean read length, Phred quality scores, Q20 and Q30 ratios, mean quality score, total read counts, and total bases sequenced, indicating high sequencing quality across all samples.
