# Supplementary material for: Direct Chemical Reprogramming of Human Fibroblasts into Retinal Progenitor-like Cells for Ocular Delivery
Source: J Funct Biomater. 2026 May 8;17(5):236. doi: 10.3390/jfb17050236 (PMC13208236; doi:10.3390/jfb17050236)
Supplement: Supplementary file 1 [file jfb-17-00236-s001.zip › Figure S3.pdf]

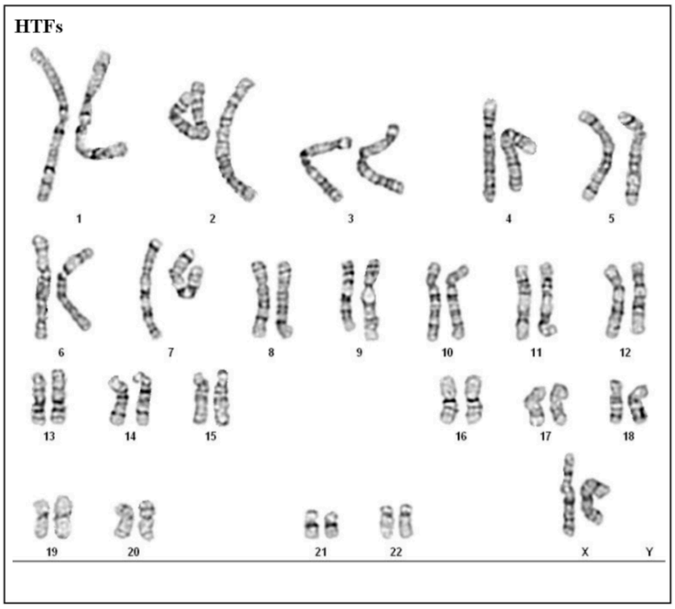

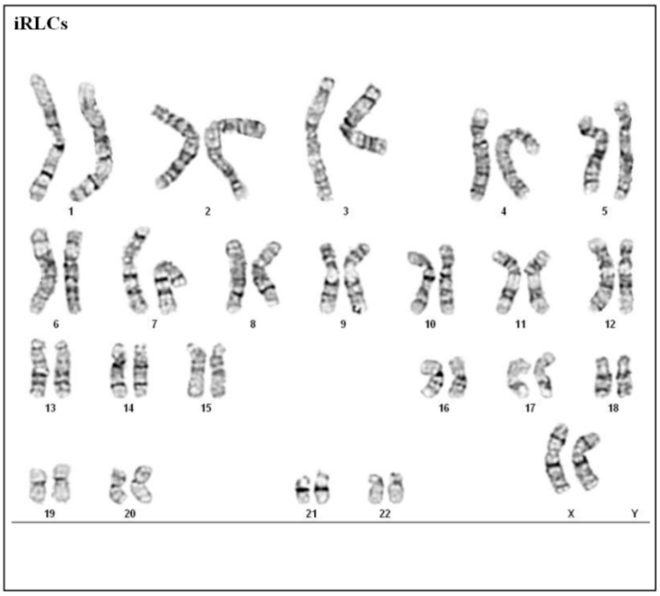

Figure S3. Karyotyping of HTFs and iRLCs. Normal female chromosomes, represented by 46,XX, were found in HTFs and pairing iRLCs. There was no abnormal chromosome number or abnormal chromosome structural balance/imbalance, such as balanced translocation, balanced inversion, duplication, deletion, unbalanced translocation, etc. Images are representative of independent biological replicates derived from different donors (n = 6).
