# Supplementary material for: Direct Chemical Reprogramming of Human Fibroblasts into Retinal Progenitor-like Cells for Ocular Delivery
Source: J Funct Biomater. 2026 May 8;17(5):236. doi: 10.3390/jfb17050236 (PMC13208236; doi:10.3390/jfb17050236)
Supplement: Supplementary file 1 [file jfb-17-00236-s001.zip › Figure S4.pdf]

A

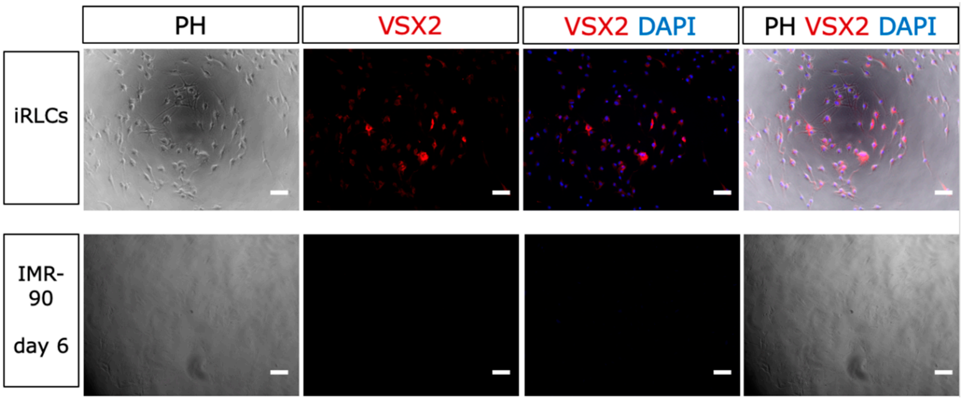
PH
VSX2
VSX2 DAPI
PH VSX2 DAPI
iRLCs
IMR-90
day 6

B

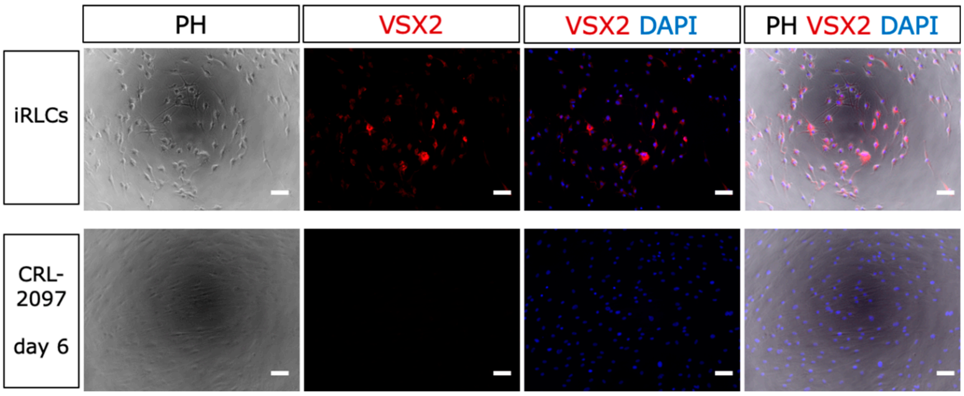
PH
VSX2
VSX2 DAPI
PH VSX2 DAPI
iRLCs
CRL-2097
day 6

C

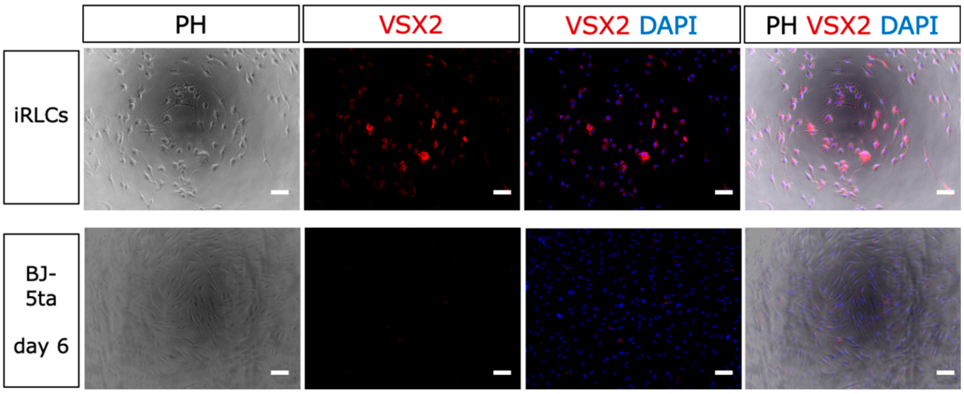
PH
VSX2
VSX2 DAPI
PH VSX2 DAPI
iRLCs
BJ-5ta
day 6

D

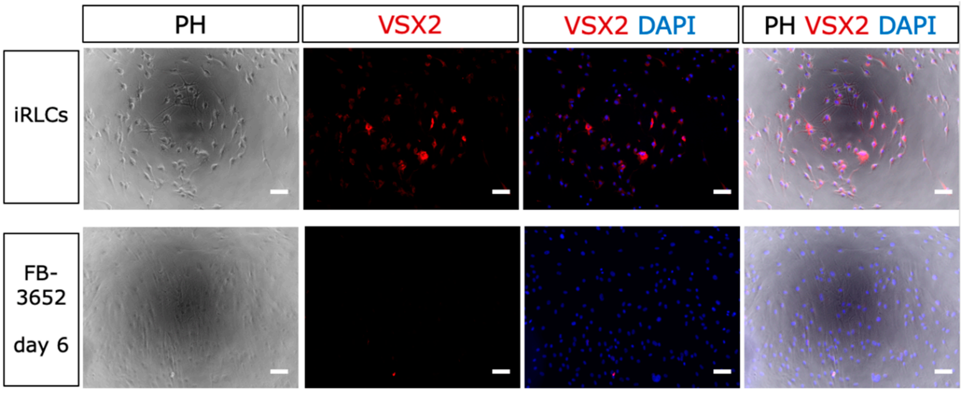
PH
VSX2
VSX2 DAPI
PH VSX2 DAPI
iRLCs
FB-3652
day 6

Figure S4. VSX2 expression following reprogramming in different fibroblast sources. Immunofluorescence staining for VSX2 at day 6 following application of the 6C reprogramming protocol. All cell types were subjected to identical reprogramming conditions. For each comparison, HTF-derived induced retinal lineage–like cells (iRLCs) are shown in the upper panels, and the corresponding non-ocular fibroblast line is shown in the lower panels. **A.** iRLCs and IMR-90 human fetal lung fibroblasts. **B.** iRLCs and CRL-2097 human neonatal foreskin fibroblasts. **C.** iRLCs and BJ-5ta human neonatal foreskin fibroblasts. **D.** iRLCs and FB-3652 human adult dermal fibroblasts. VSX2 expression was detected in HTF-derived induced cells but not in non-ocular fibroblast lines under the conditions tested. PH: phase contrast. Scale bar: 100 μm. Images are representative of cultures derived from six independent donors (n = 6).
