# Supplementary material for: Direct Chemical Reprogramming of Human Fibroblasts into Retinal Progenitor-like Cells for Ocular Delivery
Source: J Funct Biomater. 2026 May 8;17(5):236. doi: 10.3390/jfb17050236 (PMC13208236; doi:10.3390/jfb17050236)
Supplement: Supplementary file 1 [file jfb-17-00236-s001.zip › Figure S5.pdf]

A

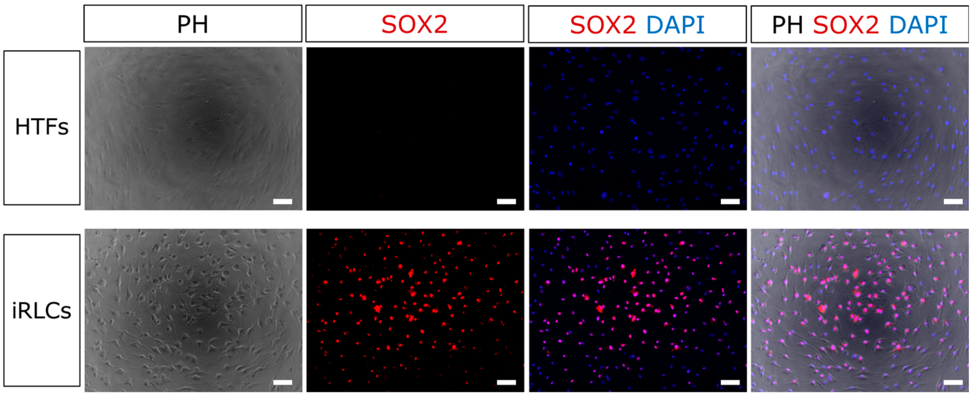
PH
SOX2
SOX2 DAPI
PH SOX2 DAPI
HTFs
iRLCs

B

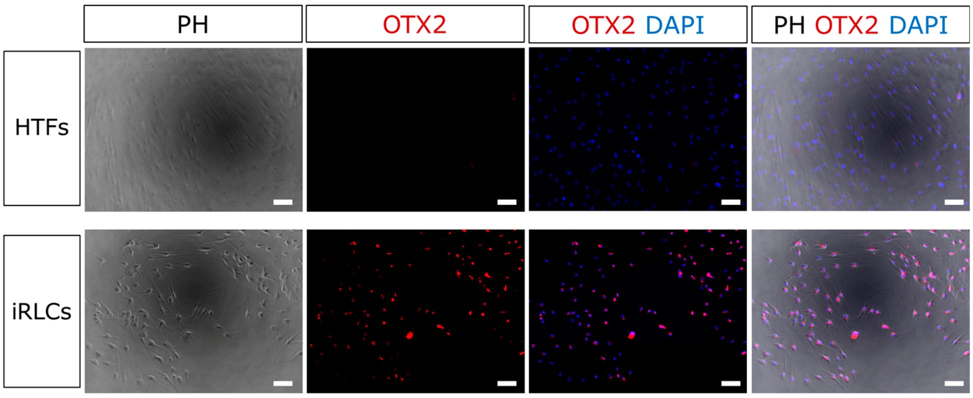
PH
OTX2
OTX2 DAPI
PH OTX2 DAPI
HTFs
iRLCs

C

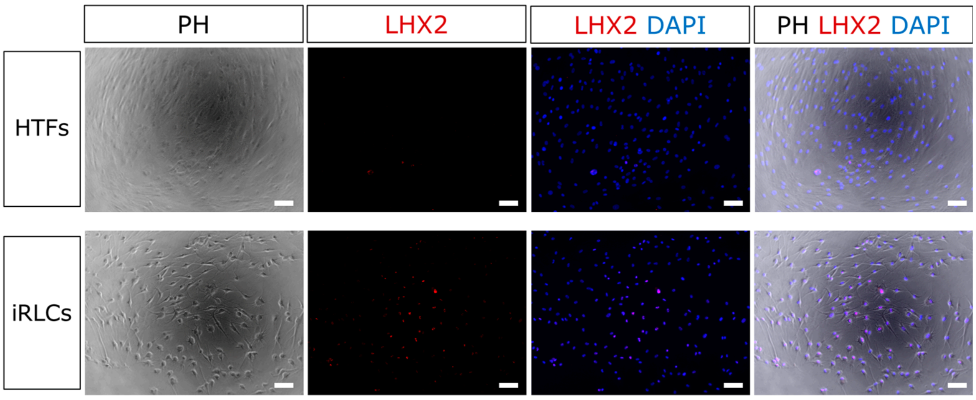
PH
LHX2
LHX2 DAPI
PH LHX2 DAPI
HTFs
iRLCs

D

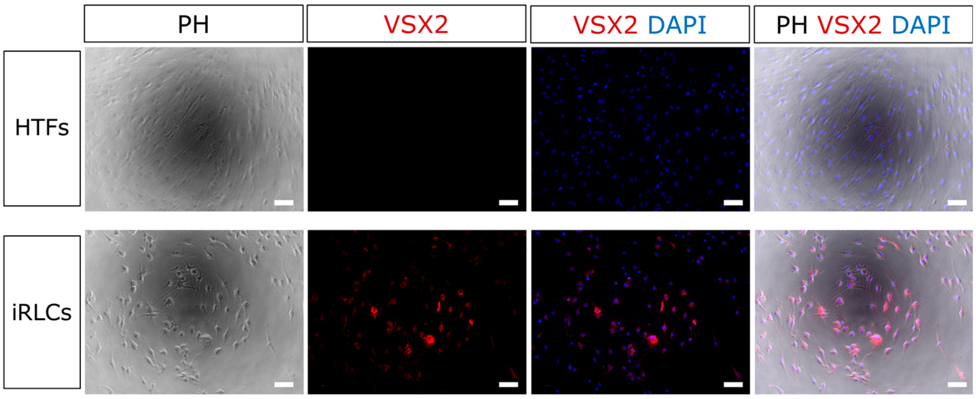

E

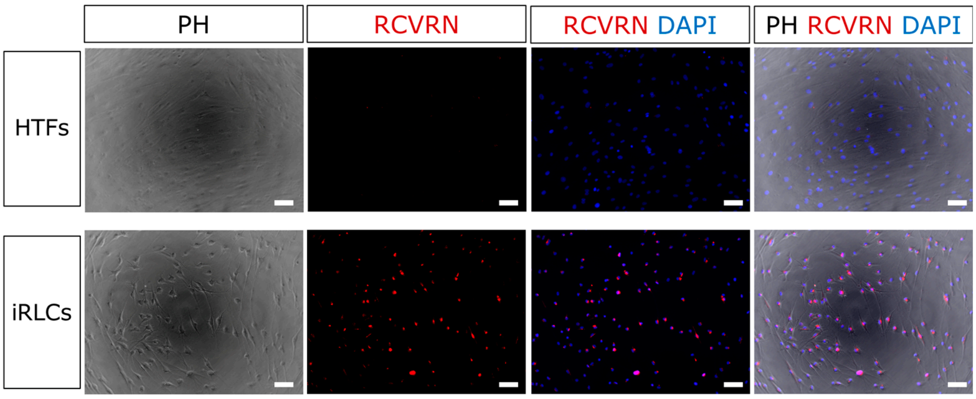

Figure S5. Immunofluorescence analysis of neural and retinal lineage–associated protein expression in HTFs and iRLCs. A–E. Representative phase-contrast (PH) and immunofluorescence images showing expression of SOX2 (A), OTX2 (B), LHX2 (C), VSX2 (D), and RCVRN (E) in parental HTFs and day 6 iRLCs following 6C reprogramming. Red fluorescence indicates target protein expression; nuclei were counterstained with DAPI (blue). Merged images are shown in the rightmost panels. Scale bar: 100 µm. Images are representative of cultures derived from six independent donors (n = 6).
