# Supplementary material for: Direct Chemical Reprogramming of Human Fibroblasts into Retinal Progenitor-like Cells for Ocular Delivery
Source: J Funct Biomater. 2026 May 8;17(5):236. doi: 10.3390/jfb17050236 (PMC13208236; doi:10.3390/jfb17050236)
Supplement: Supplementary file 1 [file jfb-17-00236-s001.zip › Figure S8.pdf]

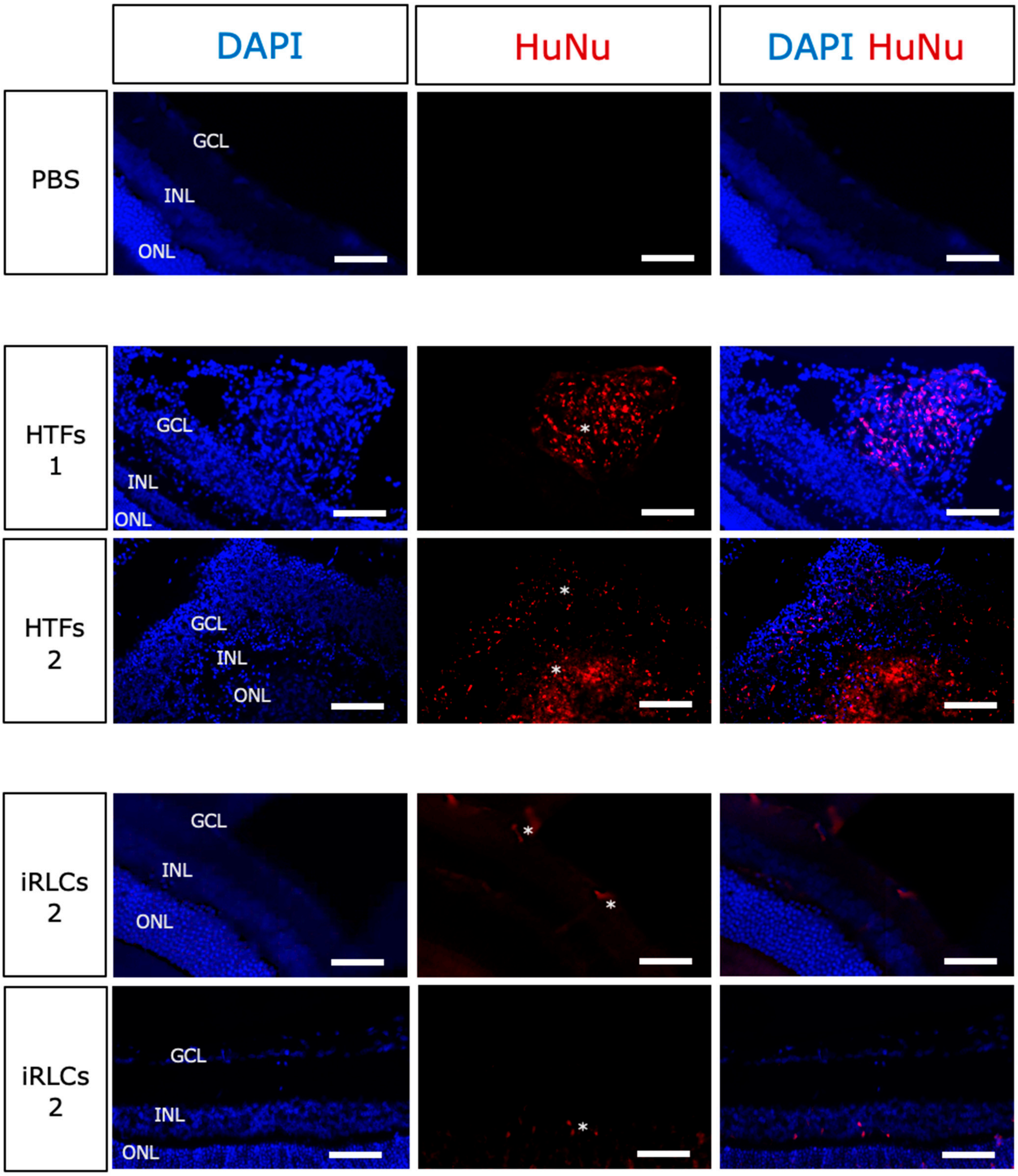

Figure S8. Additional representative immunofluorescence images from independent animals following intravitreal transplantation. DAPI (blue) and HuNu (red) staining are shown for PBS-, HTF-, and iRLC-treated eyes. Asterisks (*) indicate representative HuNu-positive signals. Retinal layers are annotated for orientation, including the GCL, INL, and ONL. Additional

sections from independent animals demonstrate minimal HuNu-positive signal in PBS-treated eyes, prominent accumulation of HuNu-positive cells in epiretinal membrane–like structures in HTF-transplanted eyes, and sparse, localized HuNu-positive signals in iRLC-transplanted eyes. Images are representative of the overall distribution patterns observed across animals and sections. Scale bars: 50 μm.
