# Supplementary material for: Direct Chemical Reprogramming of Human Fibroblasts into Retinal Progenitor-like Cells for Ocular Delivery
Source: J Funct Biomater. 2026 May 8;17(5):236. doi: 10.3390/jfb17050236 (PMC13208236; doi:10.3390/jfb17050236)
Supplement: Supplementary file 1 [file jfb-17-00236-s001.zip › Figure S9.pdf]

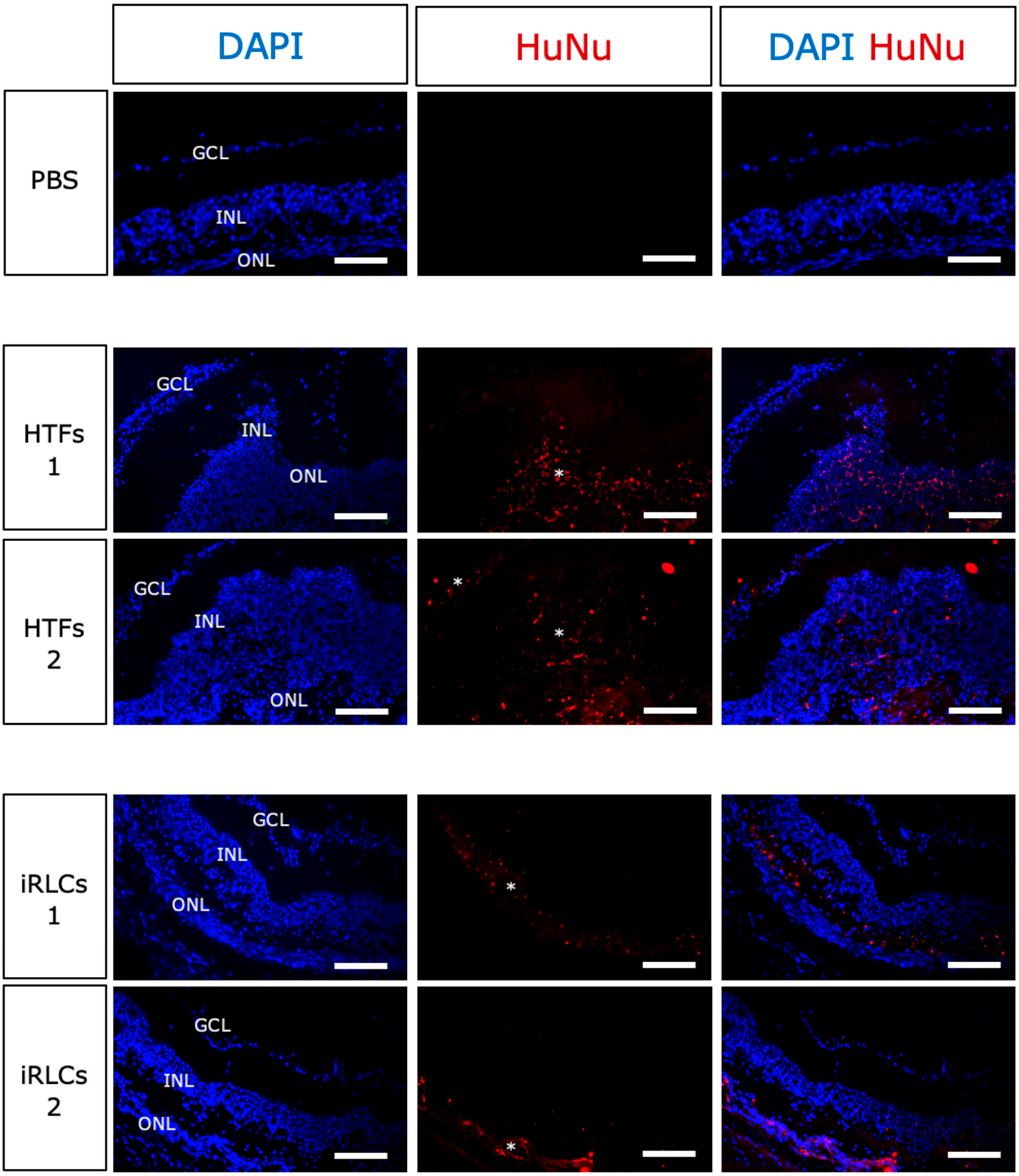

Figure S9. Additional representative immunofluorescence images from independent animals following subretinal transplantation. DAPI (blue) and HuNu (red) staining are shown for PBS-, HTF-, and iRLC-treated eyes. Asterisks (*) indicate representative HuNu-positive signals. Retinal layers are annotated for orientation, including the GCL, INL, and ONL. Additional

sections from independent animals demonstrate minimal HuNu-positive signal in PBS-treated eyes, accumulation of HuNu-positive cells in subretinal or outer retinal regions in HTF-transplanted eyes, and sparse, localized HuNu-positive signals in iRLC-transplanted eyes, primarily along the subretinal space or adjacent retinal layers. Images are representative of the overall distribution patterns observed across animals and sections. These observations reflect localized and heterogeneous signal distribution and should not be interpreted as evidence of widespread integration. Scale bars: 50 μm.
