# Supplementary material for: Direct Chemical Reprogramming of Human Fibroblasts into Retinal Progenitor-like Cells for Ocular Delivery
Source: J Funct Biomater. 2026 May 8;17(5):236. doi: 10.3390/jfb17050236 (PMC13208236; doi:10.3390/jfb17050236)
Supplement: Supplementary file 1 [file jfb-17-00236-s001.zip › Table S1.pdf]

Table S1. Summary of studies using human retinal progenitor cells on retinal diseases

Year, journal and institute	Title	Source cell	RPC markers	Methods to check gene expression	Functional analyses	Conclusion
2006<br><br>Proc Natl Acad Sci USA<br><br>University of Washington	Efficient generation of retinal progenitor cells from human embryonic stem cells.	H-1 and Hsf6 hES under retinal determination conditions for 3 weeks	● mRNA: qRT-PCR of Rx, Pax6, Lhx2, and Six3<br><br>● Protein: Immunofluorescence of Pax6, Chx10	● mRNA: qRT-PCR of Otx2, Lhx2, Pax6, Rx, Six3, Crx<br><br>● Protein: Immunofluorescence of Pax6, Chx10<br><br>● Control: Fetal RPC at 91 days	● In -vitro: Glutamate and NMDA induced calcium changes in hES cell-derived retinal neurons<br><br>Cocultures with retinal explants from both wild-type and Aipl1-/- mouse showed differentiation and integration into the mouse retina	Human ES cells can be selectively directed to a neural retinal cell fate and thus may be useful in the treatment of retinal degenerations.
2009<br><br>Exp Eye Res<br><br>Harvard Medical School	Growth kinetics and transplantation of human retinal progenitor cells.	Fetal RPC at GA 12–18 weeks	● mRNA: RT-PCR of PAX6, LHX2<br><br>● Protein: Immunofluorescence of Ki-67, nestin	● mRNA: RT-PCR of PAX6, LHX2, CRX, math5, recoverin, rhodopsin, blue opsin<br><br>● Protein: Immunofluorescence of Ki-67, nestin, rhodopsin, recoverin, red/ green opsin and blue opsin<br><br>● Control: Fetal RPC at different weeks	● In -vitro: Cocultures with retinal explants from rd1 and rhodopsin-/- mouse showed hRPCs migrated and extended their processes into the host outer nuclear layer<br><br>● In -vivo: $2*10^5$ hRPCs injected into the sub-retinal space of laser injury B6 mice and rhodopsin-/- mice, donor hRPCs survived 2 weeks post-transplantation and able to migrate, integrate and	This study is the first to generate the maximum number of cells from human fetal tissue, show that these cells remain in an undifferentiated state through late passage and that they can integrate and differentiate into mature photoreceptors both in vivo and in vitro.

					differentiate	
2009<br><br>Invest Ophthalmol Vis Sci<br><br>Harvard Medical School	Molecular characterization of human retinal progenitor cells.	Fetal RPC at GA 12–18 weeks and expanded to P1 P3 P6	● Protein:<br>Immunofluorescence of SOX2, KI67, PAX6, nestin, and vimentin<br><br>IB of CHX10, nestin, SOX2	● mRNA:<br>Microarray of CHX10, SIX6, SIX3, DACH1, OTX2, GNL3, HES5, HS1, SOX2, PAX6, NES, KI67, VIM, RPS27A<br><br>qRT-PCR of DACH1, SOX2, OTX2, GNL3, HES5, NES, PAX6, SIX6, KI67, VIM, RPS27A<br><br>● Protein:<br>Immunofluorescence of blue opsin, CRX, GFAP, KI67, nestin, PAX6, recoverin, SOX2, vimentin<br><br>IB of β-actin, CHX10, nestin, SOX2<br><br>● Control:<br>Fetal RPC at P0	● In -vitro:<br>Cocultures with retinal explants from postnatal mouse showed a significant increase of gene expression<br><br>Glutamate and NMDA induced intracellular calcium dynamics	Fetal-derived hRPCs show molecular characteristics were indicative of a retinal progenitor state up to P6.<br><br>These cells are functional, respond to excitatory neurotransmitters, and exhibit changes in expression patterns in response to co-culture with mouse retina.
2012<br><br>Eur J Neurosci<br><br>University of Toronto	Generation and clonal isolation of retinal stem cells from human embryonic stem cells.	H9 hES differentiated into pigmented colonies after 4 weeks	● Protein:<br>Immunofluorescence of Pax6, Chx10,	● mRNA:<br>qRT-PCR of Rx, Pax6, Chx10, Crx, Nrl, and rhodopsin<br><br>● Protein:<br>Immunofluorescence of rhodopsin, Pax6, Chx10, bestrophin, calbindin, Cralbp	● In -vitro:<br>Cocultures with retinal explants from B6 mice, hESC-derived RSCs can be directed toward a rod photoreceptor fate after 8 weeks<br><br>● In -vivo:<br>$5*10^4$ hESC- derived RSCs	hESC- derived RSCs emerge in an in vitro model of retinal development and are a potential source of human photoreceptors for use in transplantation.

				● Control:<br>Undifferentiated H9 hES	injected into vitreous of early postnatal mouse, 1–5% of transplanted cell survived and integrate after 3–4 weeks	
2014<br><br>Tissue Eng Part A<br><br>Harvard Medical School	Low-oxygen culture conditions extend the multipotent properties of human retinal progenitor cells.	Fetal RPC at GA 16–20 weeks under 3% O_2	● Protein:<br>Immunofluorescence of Otx2, Sox2, Pax6	● mRNA:<br>RT-PCR of Recoverin, Rhodopsin, Blue opsin, Red/green opsin, Nrl, bActin<br><br>● Protein:<br>Immunofluorescence of Otx2, Sox2, Pax6, CyclinD1, Ki67, hTERT, cMyc, Klf4, Oct4, SSEA4<br><br>IB of HIF1a, HIF2a, hTERT, Nestin, Sox2, Oct4, Klf4, and cMyc<br><br>● Control:<br>Fetal RPC under 20% O_2	● In -vitro:<br>Telomerase activity decreased with the passage in both conditions but was higher in the 3% O2 group<br><br>Following in vitro differentiation hRPCs expanded in the 3% O2 group were able to generate specialized retinal cells	Low-oxygen culture conditions act to maintain both multipotency and self-renewal properties of hRPCs in vitro.
2014<br><br>J Biol Chem<br><br>Harvard Medical School	Human retinal progenitor cell transplantation preserves vision.	GS086 hRPC line (fetal RPC at GA 16 weeks) under 3% O_2	● Protein:<br>Immunofluorescence of NeuroD1, PAX6	● mRNA:<br>qRT-PCR of NANOG, OCT4, SOX2, PAX6, SIX6, LHX2, RECOVERIN, OPSIN-1, S-OPSIN, RHODOPSIN<br><br>● Protein:<br>Immunofluorescence of β 3-Tubulin, CD44, CRX, Ki67, Nestin, NeuroD1, NRL, OTX2, PAX6, Recoverin, SSEA4,	● In -vivo:<br>$5*10^4$ hRPCs injected into subretinal space of 21-day-old RCS rats, transplanted cell arrested visual decline over time and rescued retinal morphology after 12 weeks	hRPC transplantation into the subretinal space of RCS rats was well tolerated, with no adverse effects such as tumor formation noted at 12 weeks after treatment.

				Vimentin<br><br>Flow cytometry analysis of SOX2, CD38, CD73, PAX6, HLA-A,B,C, CD133, A2B5<br><br>● Control:<br>hESCs and adult retina		
2015<br><br>J Cell Sci<br><br>Third Military Medical University	c-Kit+ cells isolated from human fetal retinas represent a new population of retinal progenitor cells.	Fetal RPC at GA 12–14 weeks, sorted by c-Kit^{+}/SS $EA4^{-}$	● Protein:<br>Immunofluorescence of Pax6, Sox2, Rax, and nestin	● Protein:<br>Immunofluorescence of c-Kit, Pax6, Sox2, Otx2, Rax, nestin, Ki67, GFAP, Thy1, recoverin<br><br>Flow cytometry analysis of Otx2, Crx, recoverin, rhodopsin, Thy1, and GFAP	● In -vivo:<br>$6*10^5$ c-Kit^{+}/$SSEA4^{-}$ hRPCs injected into subretinal space of 2–3 weeks old RCS rats, transplanted cell differentiated and survived for at least 3 months	c-Kit can be used as a surface marker for RPCs, and c-Kit+/SSEA4− RPCs exhibit the ability to self-renew and differentiate into retinal cells.
2016<br><br>Transl Vis Sci Technol<br><br>Harvard Medical School	Efficacy and safety of human retinal progenitor cells.	GS089 hRPC line (fetal RPC at GA 16 weeks) under 3% O_2	● Protein:<br>Immunofluorescence of Pax6 and Sox2	● Protein:<br>Immunofluorescence of nestin, vimentin, GFAP, βIII tubulin, Pax6 and Sox2	● In -vivo:<br>$2*10^5$ (200 K), $1*10^5$ (100 K), $5*10^4$ (50 K), and $1*10^4$ (10 K) hRPCs injected into subretinal space of 23 to 25 days old RCS rats, 28 to 29 days old WT rats and 35 to 42 days old NIH-III mice, transplanted cells integrated/ survived in dystrophic and WT rat retina up to 6 months	GS089 hRPC line appears safe and preserves retinal structure/vision at an optimal dose of 50,000 to 100,000 cells following subretinal injection into a widely used preclinical model of degenerative retinal disease.
2017	Experimental study of the biological	H1 hES differentia	● Protein:<br>Immunofluor	● mRNA:<br>qRT-PCR of Pax6, Sox2, Rax,	● In -vivo:<br>$6*10^5$ hESCs-derived RPCs	hESCs-derived RPCs could delay

Sci Rep<br><br>Zhengzhou University	properties of human embryonic stem cell-derived retinal progenitor cells.	ted into RPCs after 20 days	escence of Pax6, Sox2, Rax, Nestin	Nestin, Otx2, Crx and Recoverin<br><br>● Protein:<br>Immunofluorescence of Ki67, Pax6, Sox2, Rax, Nestin<br><br>IB of Pax6, Sox2, Rax, Nestin, Crx, recoverin<br><br>Flow cytometry analysis of Pax6, Sox2, Rax, Nestin, Otx2, Crx, recoverin<br><br>● Control:<br>Undifferentiated H1 hES	injected into subretinal space of 3 weeks old RCS rats, transplanted cell survived, Integrated, and delayed retinal degeneration for 8 weeks	the degeneration of the retina and partially restore visual function.
2017<br><br>Sci Rep<br><br>Third Military Medical University	Combined transplantation of human mesenchymal stem cells and human retinal progenitor cells into the subretinal space of RCS rats.	Fetal RPC at GA 11–13 weeks<br><br>Bone marrow hMSCs at 20–30 years old	● Protein:<br>Immunofluorescence of Pax6, Sox2, Nestin	● Protein:<br>Immunofluorescence of Ki67, Pax6, Sox2, Nestin, and GFAP<br><br>Flow cytometry analysis of Ki67, Pax6, Sox2, Nestin, and GFAP	● In -vivo:<br>$2*10^5$ hRPCs and $2*10^5$ hBMSCs injected into subretinal space of 3 weeks old RCS rats, transplanted cell survived, integrated, differentiated and delayed retinal degeneration for 12 weeks	Combining the transplantation of hRPCs and hBMSCs is a more effective strategy in stem cell-based therapy for retinal degenerative diseases.
2017<br><br>Stem Cell Res Ther<br><br>Third	Long-term safety of human retinal progenitor cell transplantation in retinitis pigmentosa patients.	Fetal RPC at GA 12–16 weeks	● Protein:<br>Immunofluorescence of Pax6, Sox2, Nestin	● mRNA:<br>qRT-PCR of Nanog, OCT4, PAX6, Six6, Crx, and recoverin<br><br>● Protein:<br>Immunofluorescence of PAX6,	● In -vitro:<br>Differentiation of hRPCs into photoreceptors by adding retinoic acid<br><br>● In -vivo:	The study for the first time confirmed the long-term safety and feasibility of vision repair by stem cell therapy in

Military Medical University				CRX, Nestin, Sox2, GFAP<br><br>Flow cytometry analysis of Nestin, PAX6, SOX2, and GFAP<br><br>● Control:<br>Undifferentiated H1 hES	$1*10^5$ hRPCs injected into subretinal space of 30 days old RCS rats, moderate recovery of vision and maintenance of the outer nuclear layer thickness after 6 weeks<br><br>$1*10^6$ hRPCs injected into subretinal space of eight adult patients diagnosed with retinitis pigmentosa, a significant improvement of visual acuity in five patients and an increase in retinal sensitivity of pupillary responses in three of the eight patients between 2 and 6 months after the transplant, but this improvement did not appear by 12 months	patients blinded by retinitis pigmentosa
2017<br><br>Tissue Eng Regen Med<br><br>Korea University College of Medicine	Generation of retinal progenitor cells from human induced pluripotent stem cell-derived spherical neural mass.	Human iPSCs differentiated into RPCs after 47 days	● Protein:<br>Immunofluorescence of CHX10, PAX6	● mRNA:<br>qRT-PCR of OCT4, FOXG1, LHX2, PAX6, SIX3, SIX6. MASH1, NEUROD1, MATH5, BRN3B, MITF, CRX, RCVRN<br><br>● Protein:<br>Immunofluorescence of SSEA-4, OCT4, Tra-1-60, Tra-1-81, CHX10, PAX6, OPN1SW	● In -vivo:<br>$5*10^4$ hiPSC-derived RPCs injected into subretinal space of 8-week-old B6 mouse, transplanted cell survived, Integrated and differentiated after 3 months	The development of RPCs using SNMs may offer a fast and useful method for neural retinal cell differentiation.

				● Control:<br>hiPSC-derived spherical neural mass (SNM)		
2018<br><br>Stem Cells Dev<br><br>Shanghai Medical College, Fudan University	Transplantation of retinal progenitor cells from optic cup-like structures differentiated from human embryonic stem cells in vitro and in vivo generation of retinal ganglion-like cells.	H9 hES differentiated into optic cup-like structures after 6 weeks	● Protein:<br>Immunofluorescence of Math5, Pax6	● Protein:<br>Immunofluorescence of Math5, Brn3b, Brn3, Pax6, Nanog, Oct 3/4 and Tuj1	● In -vivo:<br>$2*10^4$ hESC- derived cells from optic cup-like structures injected into vitreous of NMDA-treated mouse, transplanted cell integrated into the host GCL and differentiated after 4-5 weeks	RPCs can be used as an ideal source in supplying countless RGC and ESC-based replacement therapies may be a promising treatment to restore vision in patients with degenerative retinal diseases.
2018<br><br>Exp Eye Res<br><br>University of California Irvine	Sheets of human retinal progenitor transplants improve vision in rats with severe retinal degeneration.	Human fetal eyes (11–15.7 weeks post-conception)	● Protein:<br>Immunofluorescence of Chx10	● Protein:<br>Immunofluorescence of Chx10 (VSX2), NBL, MAP2, NeuN, RAX, OTX2, OTX2, CRALBP, Vimentin	● In -vivo:<br>0.7 mm × 1.2 mm of RPC sheet injected into subretinal space of P26–31 RD nude rat	Transplantation of human fetal retinal sheets could improve visual function in this rat model of advanced stage of retinal degeneration.
2019<br><br>Nat Commun<br><br>Third Military Medical University	Organoid-derived c-Kit^+/$SSEA4^-$ human retinal progenitor cells promote a protective retinal microenvironment during	c-Kit^+/$SSEA4^-$ hRPCs from hESC-derived retinal organoids	● Protein:<br>c-Kit^+/$SSEA4^-$ cells determined by flow cytometry analysis	● mRNA:<br>Transcriptome of c-Kit^+ cells from 30-, 45-, and 60-day hESC-derived retinal organoids	● In -vivo:<br>$2*10^5$ c-Kit^+/$SSEA4^-$ hRPCs injected into subretinal space of P 21 RCS rats and P7 rd1 mice	Organoid-derived c-Kit^+/$SSEA4^-$ RPCs significantly improve vision and preserve the retinal structure.

	transplantation in rodents.					
2020<br><br>Med Sci Monit<br><br>He University, Shenyang	Intravitreal injection of human retinal progenitor cells for treatment of retinal degeneration.	hRPCs from donated neural retinas of 16 to 18 weeks gestational age	● Protein:<br>Flow cytometry analysis of Nestin, Ki67, Pax6, and Chx10	● Protein:<br>Immunofluorescence of Map2, GFAP, NF, recoverin, and rhodopsin	● In -vivo:<br>Intravitreal injection of $2*10^5$ hRPCs into eyes of P 21 RCS rats	Intravitreal injection of hRPCs is effective and safe in protecting photoreceptor cells in RCS rats, but no longer effective at 12 weeks after transplantation.
2021<br><br>Stem Cells Int<br><br>University of California Irvine	Age-related macular degeneration transmitochondrial cybrids protected from cellular damage and death by human retinal progenitor cells.	hRPCs from fetal eyes (17-20 weeks gestational age)	● mRNA:<br>TaqMan assay for glial lineage (GFAP), neuronal lineage (MAP2), and neuroprotection (MDK, PTN, and FGF2)	● mRNA:<br>qRT-PCR of apoptosis (BAX, CASP3, CASP7, and CASP9), autophagy (ATG5, ATG12, LAMP2, LC3B, and PARK2), endoplasmic reticulum stress (DDIT3 and XBP1), antioxidant (GPX3, SOD2, and NQO1), and mitochondrial replication (POLG, POLRMT, and TFAM)	● In -vitro:<br>$3*10^5$ of hRPCs cocultured with AMD or age-matched normal cybrid cells to characterize the effects of hRPCs in protecting AMD cybrids from cellular and mitochondrial damage and death	hRPCs release trophic factors that protect the cybrids against the cellular and mitochondrial changes involved in AMD pathogenesis. And AMD cybrids upregulate the release of these neuroprotective factors by hRPCs while promoting hRPC differentiation.
2022<br><br>Stem Cell Res Ther	Human primitive mesenchymal stem cell-derived retinal progenitor cells	pMSCs isolated from human	● Protein:<br>Flow cytometry analysis of	● mRNA:<br>Transcriptome of pMSCs and RPCs<br><br>● Protein:	● In -vivo:<br>$2*10^5$of pMSCs or RPCs labeled with PKH26 intravitreally injected into rd12	RPCs migrated to the neural layers of the retina, where they improved the

Oakland University	improved neuroprotection, neurogenesis, and vision in rd12 mouse model of retinitis pigmentosa.	umbilical cord tissue, and differentiated towards RPCs	RCVRN	Immunofluorescence of MSC (CD90), neural (TUJ1, NESTIN, and PAX6), and retinal (RCVRN, CRX, and RHO)	mice	thickness of the respective layers and expressed cell-specific markers.
2024<br><br>Stem Cell Reports<br><br>RIKEN Center for Biosystems Dynamics Research	Label-free enrichment of human pluripotent stem cell-derived early retinal progenitor cells for cell-based regenerative therapies.	Human iPSCs differentiated into retinal organoid sheet, and isolated RPCs using a label-free ghost cytometry-based sorting system	● Protein: Flow cytometry analysis Rax::Venus-positive RPCs	● Protein: Immunofluorescence of VSX2, PAX6, RAX, CRX and BRN3	● In -vivo: Human iPSC-derived LF-GC-based-sorted retinal spheroids transplanted into the subretinal space of 6-month-old SD-Foxn1 Tg(S334ter)3LavRrrc nude rats	The sorted cells reproducibly formed retinal spheroids large enough for transplantation and developed mature photoreceptors in the retinal degeneration rats.
