# Supplementary material for: Direct Chemical Reprogramming of Human Fibroblasts into Retinal Progenitor-like Cells for Ocular Delivery
Source: J Funct Biomater. 2026 May 8;17(5):236. doi: 10.3390/jfb17050236 (PMC13208236; doi:10.3390/jfb17050236)
Supplement: Supplementary file 1 [file jfb-17-00236-s001.zip › Table S4.pdf]

Table S4. Summary of studies on transplantation of RPCs in RCS rats

Year & journal	Institution	Title	RCS rat numbers	Timing	ERG light intensity	ERG amplitude
2014<br>J Biol Chem<br>(IF = 4.011)	Harvard Medical School	Human Retinal Progenitor Cell Transplantation Preserves Vision	● Group A<br>2 µl of hRPC cell suspension (approximately 5×10^4 cells)<br>● Group B<br>Hanks' balanced salt solution–N-acetylcysteine<br>● Group C<br>baseline control without subretinal injection	● 12 weeks postoperative	● Scotopic 0.01 and 0.3 cd s/m2<br>● Photopic 30 cd s/m2	● b-waves
2015<br>J Cell Sci<br>(IF = 4.401)	Third Military Medical University	c-Kit$^+$ cells isolated from human fetal retinas represent a new population of retinal progenitor cells	● Group A<br>3 µl of a c-Kit$^+$/SSEA4$^-$ cell suspension (cell concentration, 2×10^5 cells/µl)<br>● Group B<br>3 µl of HBSS	● 4, 8 and 12 weeks postoperative	● 0.3 cds−1 m−2 and 3.0 cds−1 m−2	● scotopic b-waves
2017<br>Sci Rep<br>(IF = 4.122)	Zhengzhou University	Experimental Study of the Biological Properties of Human Embryonic Stem Cell–Derived Retinal Progenitor	● Group A<br>3-µL suspension solution containing approximately 6×10^5 differentiated cells<br>● Group B<br>3-µL HBSS	● 4, 8 and 12 weeks postoperative	● unspecified	● scotopic b- wave

		Cells				
2017<br>Sci Rep<br>(IF = 4.122)	Third Military Medical University	Combined transplantation of human mesenchymal stem cells and human retinal progenitor cells into the subretinal space of RCS rats	● Group A<br>5 µl/eye, total 4 × 10^5 cells/eye of HRPCs<br>● Group B<br>5 µl/eye, total 4 × 10^5 cells/eye of HBMSCs<br>● Group C<br>5 µl/eye, containing HRPCs (2 × 10^5 cells/eye) and HBMSCs (2 × 10^5 cells/eye)<br>● Group D<br>5 µl 0.01 M PBS	● 3, 6, 9 and 12 weeks postoperative	● 3.0 cd·s/m2	● scotopic a- wave and b- wave
2017<br>Stem Cell Res Ther<br>(IF = 4.963)	Third Military Medical University	Long-term safety of human retinal progenitor cell transplantation in retinitis pigmentosa patients	● Group A<br>5 µl of a RPC suspension (1 × 10^5 cells)<br>● Group B<br>5 µl 0.01 M PBS	● 3 and 6 weeks postoperative	● 5 dB flash	● scotopic b- wave
2018<br>Int J Clin Exp Pathol<br>(IF = 1.706)	General Hospital of Chinese People's Liberation Army	Comparison of human retinal progenitor cells cultured in media with or without serum: in vitro and in vivo	● Group A<br>5 µl with total 8×10^5 cells/eye of serum-free hRPCs<br>● Group B<br>5 µl with total 8×10^5 cells/eye of	● 3 and 6 weeks postoperative	● -6.3 log cd-s. m-2 to 0.6 log cd-s. m-2	● Max-b wave and Rod-b wave

		characteristics and retinal transplantation	serum-cultured hRPCs<br>● Group C<br>5 µl PBS<br>● Group D<br>untreated			
2019<br>Nat Commun<br>(IF = 12.121)	Third Military Medical University	Organoid-derived C-Kit^{+}/SSEA4^{-} human retinal progenitor cells promote a protective retinal microenvironment during transplantation in rodents	● Group A<br>2×10^5 human organoid-derived cells in 2 µl of HBSS<br>● Group B<br>2×10^5 hRPCs in 2 µl of HBSS<br>● Group C<br>2 µl of HBSS	● 4w, 8w, 12w, and 16w postoperative	● 0.5 log (cd*s/m2)	● scotopic b-waves
