# Supplementary material for: Direct Chemical Reprogramming of Human Fibroblasts into Retinal Progenitor-like Cells for Ocular Delivery
Source: J Funct Biomater. 2026 May 8;17(5):236. doi: 10.3390/jfb17050236 (PMC13208236; doi:10.3390/jfb17050236)
Supplement: Supplementary file 1 [file Figure S6.pdf › Figure S6.pdf]

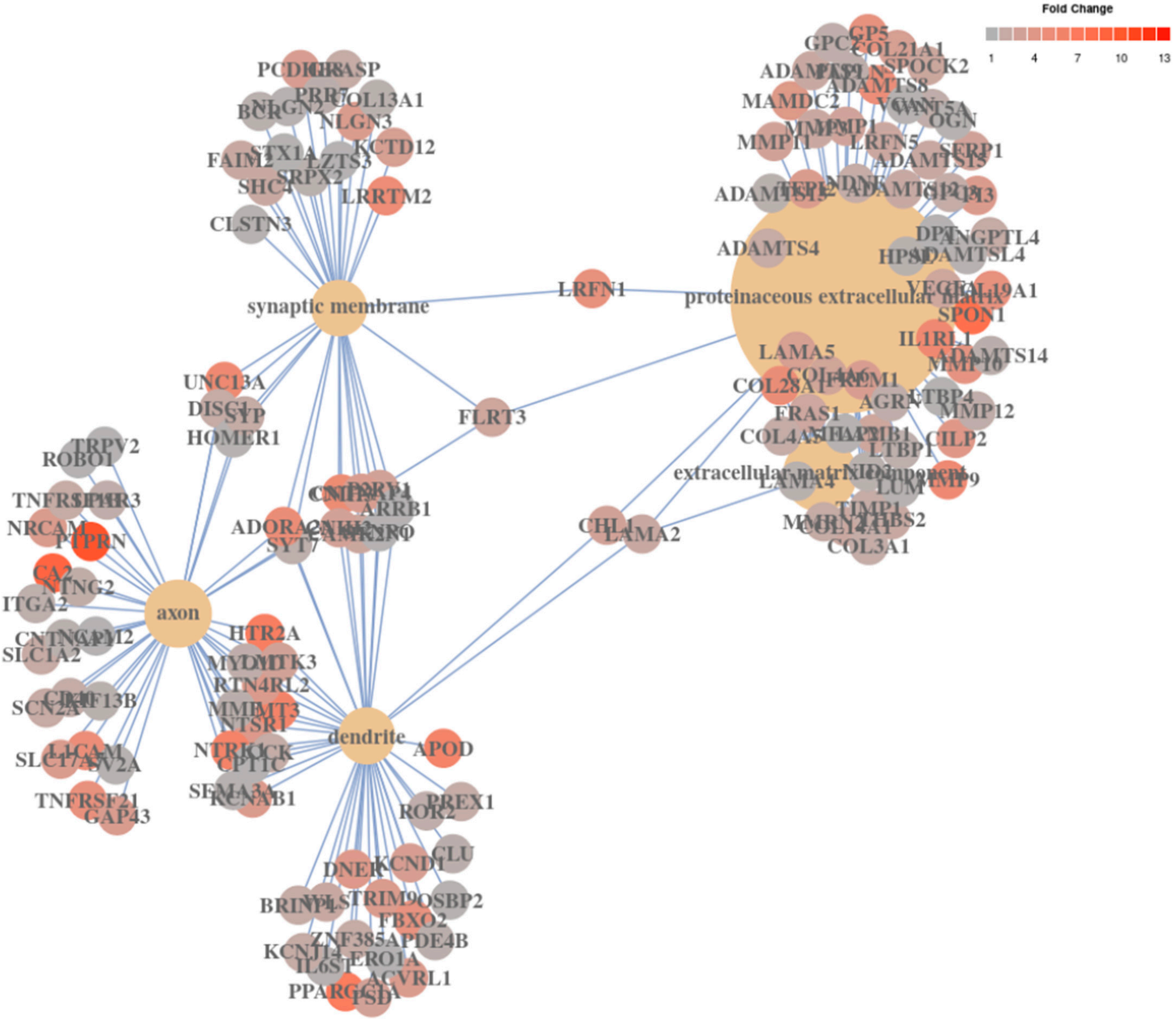

Figure S6. Cnet plot illustrating representative upregulated genes contributing to enriched biological process and cellular component categories. Node size reflects gene–category connectivity, and color intensity indicates fold change.
